# Combining Genome-Wide Association Mapping and Transcriptional Networks to Identify Novel Genes Controlling Glucosinolates in *Arabidopsis thaliana*

**DOI:** 10.1371/journal.pbio.1001125

**Published:** 2011-08-16

**Authors:** Eva K. F. Chan, Heather C. Rowe, Jason A. Corwin, Bindu Joseph, Daniel J. Kliebenstein

**Affiliations:** 1 Department of Plant Sciences, University of California–Davis, Davis, California, United States of America; 2 Monsanto Company, Vegetable Seeds Division, Woodland, California, United States of America; Georgia Institute of Technology, United States of America

## Abstract

**Background:**

Genome-wide association (GWA) is gaining popularity as a means to study the architecture of complex quantitative traits, partially due to the improvement of high-throughput low-cost genotyping and phenotyping technologies. Glucosinolate (GSL) secondary metabolites within *Arabidopsis* spp. can serve as a model system to understand the genomic architecture of adaptive quantitative traits. GSL are key anti-herbivory defenses that impart adaptive advantages within field trials. While little is known about how variation in the external or internal environment of an organism may influence the efficiency of GWA, GSL variation is known to be highly dependent upon the external stresses and developmental processes of the plant lending it to be an excellent model for studying conditional GWA.

**Methodology/Principal Findings:**

To understand how development and environment can influence GWA, we conducted a study using 96 *Arabidopsis thaliana* accessions, >40 GSL phenotypes across three conditions (one developmental comparison and one environmental comparison) and ∼230,000 SNPs. Developmental stage had dramatic effects on the outcome of GWA, with each stage identifying different loci associated with GSL traits. Further, while the molecular bases of numerous quantitative trait loci (QTL) controlling GSL traits have been identified, there is currently no estimate of how many additional genes may control natural variation in these traits. We developed a novel co-expression network approach to prioritize the thousands of GWA candidates and successfully validated a large number of these genes as influencing GSL accumulation within *A. thaliana* using single gene isogenic lines.

**Conclusions/Significance:**

Together, these results suggest that complex traits imparting environmentally contingent adaptive advantages are likely influenced by up to thousands of loci that are sensitive to fluctuations in the environment or developmental state of the organism. Additionally, while GWA is highly conditional upon genetics, the use of additional genomic information can rapidly identify causal loci en masse.

## Abstract 2

Genome-wide association mapping is highly sensitive to environmental changes, but network analysis allows rapid causal gene identification.

## Author Summary

Understanding how genetic variation can control phenotypic variation is a fundamental goal of modern biology. A major push has been made using genome-wide association mapping in all organisms to attempt and rapidly identify the genes contributing to phenotypes such as disease and nutritional disorders. But a number of fundamental questions have not been answered about the use of genome-wide association: for example, how does the internal or external environment influence the genes found? Furthermore, the simple question of how many genes may influence a trait is unknown. Finally, a number of studies have identified significant false-positive and -negative issues within genome-wide association studies that are not solvable by direct statistical approaches. We have used genome-wide association mapping in the plant *Arabidopsis thaliana* to begin exploring these questions. We show that both external and internal environments significantly alter the identified genes, such that using different tissues can lead to the identification of nearly completely different gene sets. Given the large number of potential false-positives, we developed an orthogonal approach to filtering the possible genes, by identifying co-functioning networks using the nominal candidate gene list derived from genome-wide association studies. This allowed us to rapidly identify and validate a large number of novel and unexpected genes that affect *Arabidopsis thaliana* defense metabolism within phenotypic ranges that have been shown to be selectable within the field. These genes and the associated networks suggest that *Arabidopsis thaliana* defense metabolism is more readily similar to the infinite gene hypothesis, according to which there is a vast number of causative genes controlling natural variation in this phenotype. It remains to be seen how frequently this is true for other organisms and other phenotypes.

## Introduction

Biologists across fields possess a common need to identify the genetic variation causing natural phenotypic variation. Genome-wide association (GWA) studies are a promising route to associate phenotypes with genotypes, at a genome-wide level, using “unrelated” individuals [1]. In contrast to the traditional use of structured mapping populations derived from two parent genomes, GWA studies allow a wide sampling of the genotypes present within a species, potentially identifying a greater proportion of the variable loci contributing to polygenic traits. However, the uneven distribution of this increased genotypic diversity across populations (population structure), as well as the sheer number of statistical tests performed in a genome-wide scan, can cause detection of a high rate of “false-positive” genotype-phenotype associations that may make it difficult to distinguish loci that truly affect the tested phenotype [1]–[5]. Epistasis and natural selection can also lead to a high false-negative rate, wherein loci with experimentally validated effects on the focal trait are not detected by GWA tests [4]–[5].

Repeated detection of a genotype-phenotype association across populations or experiments has been proposed to increase support for the biological reality of that association, and has even been proposed as a requirement for validation of trait-phenotype associations [2]. However, replication across populations or experiments is not solely dependent upon genotypes, but also differences in environment and development that significantly influence quantitative traits [5]–[8]. Thus, validation of a significant association through replication, while at face value providing a stringent criterion for significance, may bias studies against detection of causal associations that show significant Genotype×Environment interactions [9]. In this study we employed replicated genotypes to test the conditionality of GWA results upon the environment or development stage within which the phenotype was measured.

Integrating GWA mapping results with additional forms of genome-scale data, such as transcript profiling or proteomics datasets, has also been proposed to strengthen support for detected gene-trait associations and reduce the incidence of false-positive associations [10]. To date, network approaches have largely focused upon comparing GWA results with natural variation in gene expression across genotypes in transcriptomic datasets (i.e., expression quantitative trait loci (eQTLs)) [11]–[13]. This requires that candidate genes show natural variation in transcript accumulation, which is not always the functional level at which biologically relevant variation occurs [14]. Another network approach maps GWA results onto previously generated interaction networks within a single genotype, such as a protein-protein interaction network, enhancing support for associations that cluster within the network [15]. This network filtering approach has yet to be tested with GWA data where the environment or tissue is varied.

To evaluate the influence of environmental or developmentally conditional genetics on GWA mapping and the utility of network filtering in identifying candidate causal genes, we focused on defense metabolism within the plant *Arabidopsis thaliana*. *A. thaliana* has become a key model for advancing genetic technologies and analytical approaches for studying complex quantitative genetics in wild species [16]. These advances include experiments testing the ability of genome resequencing and transcript profiling to elucidate the genetics of complex expression traits [17]–[19] and querying the complexity of genetic epistasis in laboratory and natural populations [20]–[26]. Additionally, *A. thaliana* has long provided a model system for applying concepts surrounding GWA mapping [3]–[5],[27]–[30].

**Figure 1:**
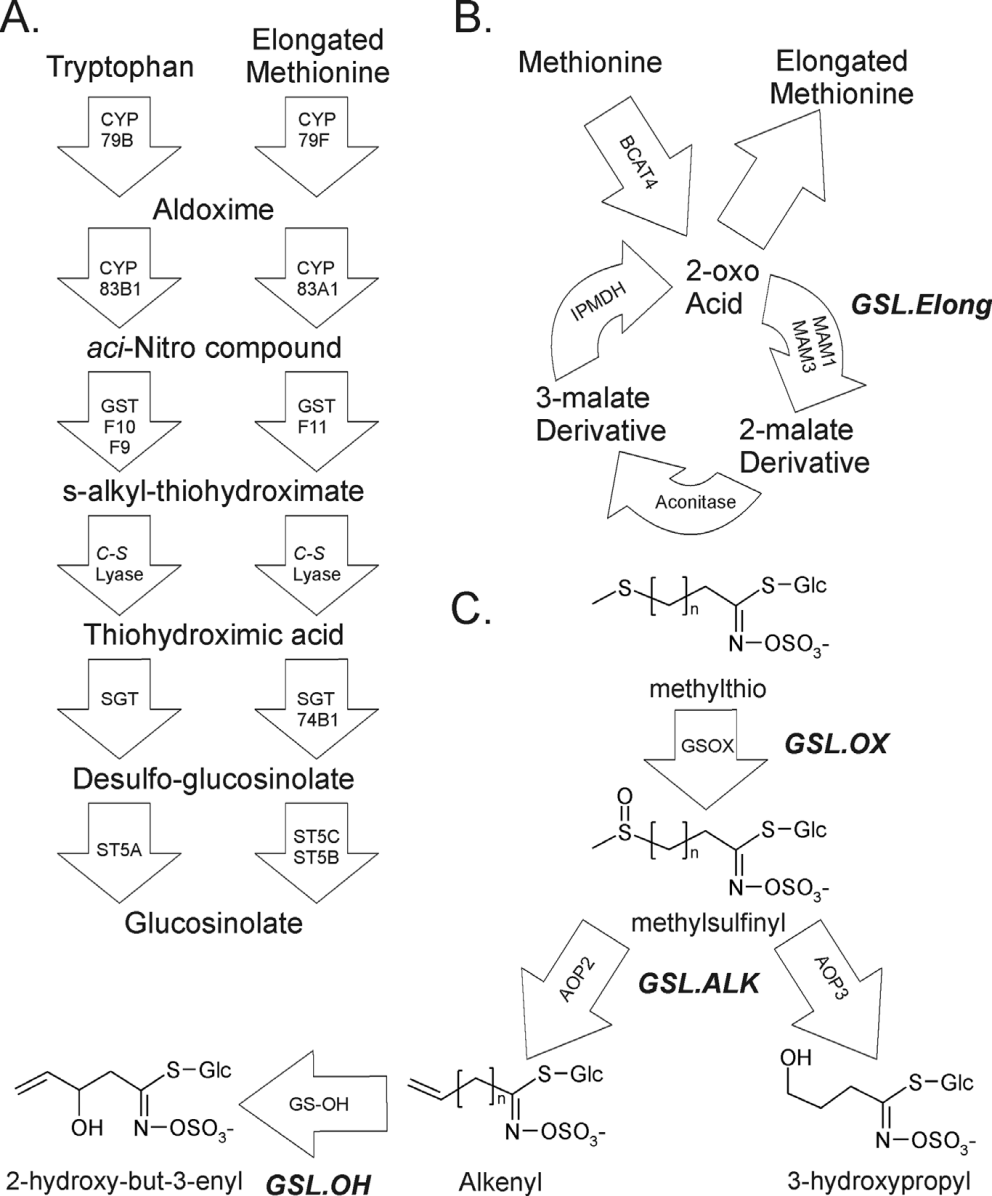
GSL Biosynthesis and Cloned QTL. Arrows show the known and predicted steps for GSL biosynthesis with the gene name for each biochemical reaction within the arrow. For compounds that are undetected intermediates, chemical names only are provided. For detected compounds, both the structure and chemical name are provided. The position of known genetic loci controlling biosynthetic variation is shown in italics. (A) The pathway and genes responsible for the production of the core GSL structure from tryptophan (indolic GSL) and methionine (aliphatic GSL). (B) The chain elongation cycle for aliphatic GSL production. Each cycle of these reactions adds a single carbon to a 2-oxo-acid, which is then trans-aminated to generate homo-methionine for aliphatic GSL biosynthesis. The *GSL.Elong* QTL alters this cycle through variation at the *MAM1*, *MAM2*, and *MAM3* genes that leads to differential GSL structure and content [71],[142]. (C) The enzymes and genetic loci controlling aliphatic GSL side chain modification within the Bay-0 × Sha RIL population. Side-chain modification is controlled by variation at the *GSL.ALK* QTL via *cis*-eQTL at the *AOP2* and *AOP3* genes. The Cvi and Sha accessions express *AOP2* to produce alkenyl GSL. In contrast, the L*er* and Bay-0 accessions express *AOP3* to produce hydroxyl GSL. Col-0 is null for both *AOP2* and *AOP3*, producing only the precursor methylsulfinyl GSL [61],[143]. The *GSL.OX* QTL appear to be controlled by *cis*-eQTL regulating flavin-monoxygenase enzymes (GS-OX1 to 5) that oxygenate a methylthio to methylsulfinyl GSL [55],[58]. The *GSL.OH* QTL is a *cis*-eQTL in the GS-OH gene which encodes the enzyme for the oxygenation reaction [56].

As a model set of phenotypes, we used the products of two related *A. thaliana* secondary metabolite pathways, responsible for aliphatic and indolic glucosinolate (GSL) biosynthesis. These pathways have become useful models for quantitative genetics and ecology (Figure 1) [31]. Aliphatic, or methionine-derived, GSL are critical determinants of fitness for *A. thaliana* and related cruciferous species via their ability to defend against insect herbivory and non-host pathogens [32]–[35]. Indolic GSL, derived from tryptophan, play important roles in resistance to pathogens and aphids [36]–[40]. *A. thaliana* accessions display significant natural genetic variation controlling the production of type and amount of both classes of GSL, with direct impacts on plant fitness in the field [33],[41]–[47]. Additionally, GSL display conditional genetic variation dependent upon both the environment and developmental stage of measurement [48]–[51]. GSL thus provide an excellent model to explore the impact of conditional genetics upon GWA analysis.

While the evolutionary and ecological importance of GSL is firmly established, the nearly complete description of GSL biosynthetic pathways provides an additional practical advantage to studying these compounds [52]–[54]. A large number of QTL and genes controlling GSL natural variation have been cloned from *A. thaliana* using a variety of network biology approaches similar to network filtering in GWA studies (Figure 1) [55]–[59]. These provide a set of positive control genes of known natural variability and importance to GSL phenotypes, enabling empirical assessment of the level of false-positive and false-negative associations.

Within this study, we measure GSL phenotypes in two developmental stages and stress conditions/treatments using a collection of wild *A. thaliana* accessions to test the relative influence of these components upon GWA. In agreement with previous analyses from structured mapping populations, we found that differences in development have more impact on conditioning genetic variation in *A. thaliana* GSL accumulation. This is further supported by our observation that GWA-identified candidate genes show a non-random distribution across the three datasets with the GWA candidates from the two developmental stages analyzed overlapping less than expected. The large list of candidate genes identified via GWA was refined with a network co-expression approach, identifying a number of potential networks. A subset of loci from these networks was validated for effects on GSL phenotypes. Even for adaptive traits like GSL accumulation, these analyses suggest the influence of numerous small effect loci affecting the phenotype at levels that are potentially exposed to natural selection.

## Results

### GSL Analysis

We measured GSL from leaves of 96 *A. thaliana* accessions at 35 d post-germination [27]–[28] using either untreated leaves or leaves treated with AgNO_3_ (silver) to mimic pathogen attack. In addition, we measured seedling glucosinolates from the same accessions to provide a tissue comparison as well as a treatment comparison. Seedlings were measured at 2 d post-germination at a stage where the GSL are largely representative of the GSL present within the mature seed [48],[60]. GSL from both foliar and seedling tissue grown under these conditions have been measured in multiple independent QTL experiments that used recombinant inbred line (RIL) populations generated from subsets of these 96 accessions, thus providing independent corroboration of observed GSL phenotypes [41],[51],[61]. For the untreated leaves, this analysis detected 18 aliphatic GSL compounds and four indolic GSL compounds. These combined with an additional 21 synthetic variables that describe discrete components of the biochemical pathway to total 43 GSLtraits for analysis [4],[61]–[62]. For the AgNO_3_-treated samples, we detected only 16 aliphatic GSL and four indolic GSL, but also were able to measure camalexin, which is related to indolic GSL (Table S3), which in combination with derived measures provided us with 42 AgNO_3_ treated GSL traits [61]. For the seedling GSL samples, we detected 19 aliphatic GSLs, two indolic, and three seedling specific phenylalanine GSLs (Table S4), which in combination with derived descriptive variables gave us a total of 46 total GSL traits [61].

### Genetic, Environmental, and Developmental Effects on GSL

**Figure 2:**
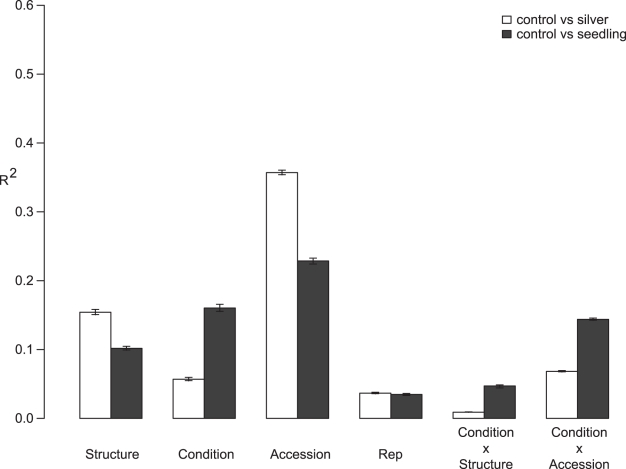
Analysis of variance of glucosinolates. The proportion of total variance (R^2^) is shown for the different model terms using a nested ANOVA where Accession is nested within Structure. The control and each of the conditional datasets—AgNO_3_-treated (white) or seedling (black)—were combined to test the effect of the specific condition upon the partitioning of variance across accession, population structure, and environmental or developmental conditions. The interaction terms are also shown.

Population stratification has previously been noted in this set of *A. thaliana* accessions, where eight subpopulations were proposed to describe the accessions' genetic differences [27]–[28]. Less explored is the joint effect of population structure and environmental factors, both external (exogenous treatment) and internal (tissue comparison) on GSL. We used our three glucosinolate datasets to test for potential confounding effects of environmental variation, population structure, and their various interaction terms upon the GSL phenotypes (Figure 2). On average, 36% (silver versus control) and 23% (seedling versus control) of phenotypic variance in GSL traits was solely attributable to accession. An additional 7% (silver versus control) and 14% (seedling versus control) of phenotypic variance was attributable to an interaction between accession and treatment or tissue. This suggests that, on average and given the statistical power of the experiments, 30%–50% of the detectable genetically controlled variance is stable across conditions, while at least 20% of the variance is conditional on treatment and/or tissue.

In contrast, population structure by itself accounted for 10%–15% of total variance in GSL (Figure 2). Interestingly, significantly less variance (<5%) could be attributed to interaction of treatment or tissue with population structure. This suggests that for GSL, large-effect polymorphisms that may be linked with population structure are stable across treatment and tissue while the polymorphisms with conditional effects are less related to the species demographic structure (Figure 2). This is consistent with QTL studies using RIL that find greater repeatability of large-effect QTL across populations and conditions than of treatment-dependent loci [41],[51],[61],[63]. This is further supported by the fact that we utilized replication of defined genotypes across all conditions and tissues and as such have better power to detect these effects than in systems where it is not possible to replicate genotypes. As such, controlling for population structure will reduce the number of false-positives detected but lead to an elevated false-negative rate, given this significant association between the measured phenotypes and population structure.

Interestingly, developmental effects (average of 15%) accounted for 3 times more of the variation in GSL than environmental effects (average 5%). In particular, only three GSL (two indolic GSL, I3M and 4MOI3M, and total indolic GSL) were affected more strongly by AgNO_3_-treatment than by accession (Table S1 and Figure S1), whereas 11 GSL traits were found to be influenced more by tissue type than accession (Table S2). This agrees with these indolic GSL being regulated by defense response [36],[64]. Similarly, twice as much GSL variation could be attributed to the interaction between accession and tissue type compared to the interaction between accession and AgNO_3_ treatment. Thus, it appears that intraspecific genetic variation has greater impact on GSL in relation to development than in response to simulated pathogen attack.

### Genome-Wide Association Study

Using 229,940 SNP available for this collection of 96 accessions, we conducted GWA-mapping for GLS traits in both the Seedling and Silver datasets using a maximum likelihood approach that accounts for genetic similarity (EMMA) [65]. This identified a large number of significant SNPs and genes for both datasets (Table 1). We tested the previously published criteria used to assess significance of candidate genes to ensure that different treatments or tissues did not bias the results produced under these criteria [4]. These criteria required ≥1 SNP, ≥2 SNPs, or ≥20% of SNPs within a gene to show significant association with a specific GSL trait. This test was independently repeated for all GSL traits in both datasets (Tables S5 and S6). As previously found using the control leaf GSL data, the more stringent ≥2 SNPs/gene criterion greatly decreased the overall number of significant genes identified while not overtly influencing the false-negative rate when using a set of GSL genes known to be naturally variable and causal within the 96 accessions (Tables 2 and 3). Interestingly, including multiple treatments and tissues did not allow us to decrease the high empirical false-negative rate (∼75%) in identifying validated causal candidate genes (Table 3) [4],[31]. Using the ≥2 SNPs/gene criterion identified 898 genes for GSL accumulation in silver-treated leaves and 909 genes for the seedling GSL data. As previously found, the majority of these candidate genes were specific to a subset of GSL phenotypes and no gene was linked to all GSL traits within any dataset (Figure S2) [4].

**Table 1:** GWA mapping summary.

GWA Descriptor	Silver	Seedling
Total # SNP tested	229,940	229,940
Total # genes tested	31,505	31,505
Avg # sig SNP per trait	230	230
Total # unique genes over all traits	898	909
Avg # sig genes per trait	37	39
Range (# genes sig per trait)	26–50	24–54
Avg # sig SNP per gene per trait	3	3
Range (Avg # sig SNP per gene per trait)	2–4	2–4
Max # sig SNP per gene per trait	7	10
Range (max # sig SNP per gene per trait)	4–16	4–13

Summary of statistical results from GWA-mapping on two different GSL datasets from 96 accessions (Silver and Seedling). # indicates the number of the items indicated, sig means the events crossing the significance threshold, and Avg is the average.

**Table 2:** Using known GSL genes to estimate thresholds in GWA mapping.

GWA Descriptor	Dataset	≥1 SNP per Gene	≥2 SNP per Gene	≥20% SNP per Gene
# of sig genes	silver	4843	898	1,025
	seedling	4767	909	1,029
GSL genes in sig genes	silver	0.6%	1.1%	0.6%
	seedling	0.4%	0.9%	0.6%
Sig GSL genes	silver	18.7%	6.7%	4.0%
	seedling	14.0%	5.3%	4.0%

Shown are the numbers of significant genes identified in the two datasets (silver and seedling) using three different call thresholds. The percentage of the significant genes that are known GSL genes is provided as well as the fraction of all known GSL genes identified at each threshold. Sig, significant.

**Table 3:** Recovery of known causal GSL genes in GWA mapping.

AGI	Name	Control	Silver	Seedling
AT1G12140	GSOX5	—	—	—
AT1G24100	UGT74B1	—	—	—
AT1G62540	GSOX2	—	—	—
AT1G62560	GSOX3	—	—	—
AT1G62570	GSOX4	Yes	—	Yes
AT1G65860	GSOX1	—	—	—
AT2G25450	GS-OH	—	—	—
AT2G31790	UGT74C1	—	—	—
AT4G03050	AOP3	Yes	Yes	Yes
AT4G03060	AOP2	Yes	Yes	Yes
AT5G07690	MYB29	—	—	—
AT5G07700	MYB76	—	—	—
AT5G23010	MAM1	Yes	Yes	Yes
AT5G57220	CYP81F2	—	—	—
AT5G60890	ATR1/MYB34	—	—	—
AT5G61420	MYB28	—	—	—

Shown are genes that have been previously shown to be both genetically polymorphic and linked to GSL accumulation within the 96 accessions for the Silver and Seedling GSL datasets as well as the previously published control leaf GWA dataset [4],[31]. Yes, if the gene has ≥2 SNPs showing significant associations to one or more GSL traits in the corresponding GWA; Dash (—), no significant associations.

We estimated the variance explained by the candidate GWA genes identified in this study using a mixed polygenic model of inheritance for each phenotype within each dataset using the GenABEL package in R [66]–[67]. This showed that, on average, the candidate genes explained 37% of the phenotypic variation with a range of 1% to 99% (Table S10). Interestingly, if the phenotypes are separated into their rough biosynthetic classes of indolic, long-chain, or short-chain aliphatic [68], there is evidence for different levels of explained phenotypic variation where indolic has the highest percent variance at 45% while short-chain has the lowest at 25% (*p* = 0.001). This is not explainable by differential heritability as the short-chain aliphatic GSLs have the highest heritability in numerous studies including this one (Tables S1 and S2) [4],[41],[61]. This is instead likely due to the fact that short-chain aliphatic GLS show higher levels of multi-locus epistasis that complicates the ability to estimate the explained variance within GWA studies [31],[41],[61].

### Treatment and Tissue Contrasts

**Figure 3:**
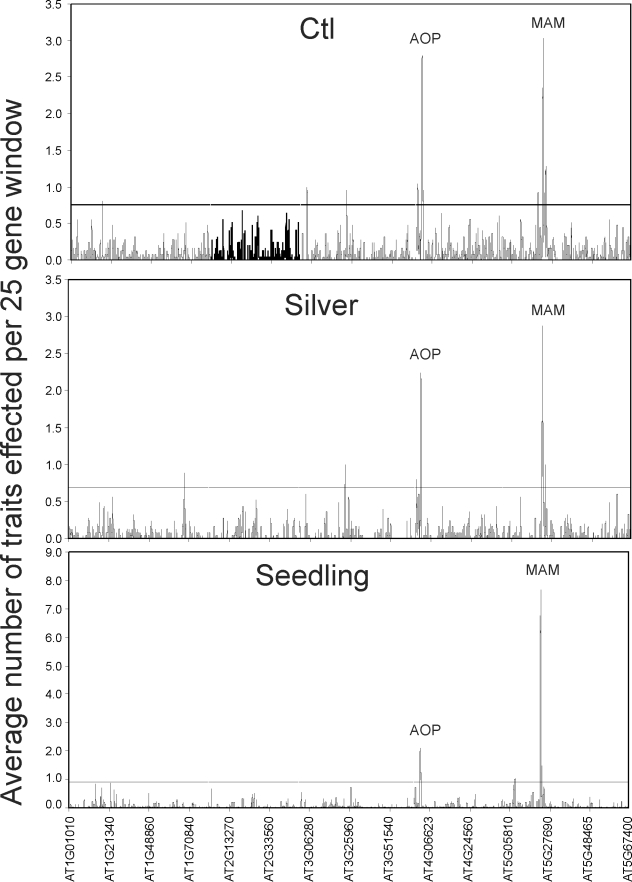
Genomic hotspots of GWA positive candidate genes. A 25-gene sliding window analysis was done to survey the genomic distribution of genes with significant associations to the different GSL traits in the three datasets. The sliding window took the average number of traits affected across every 25 genes such that a value above 1 within a 25-gene window implies each gene in the window affects one GSL trait. The horizontal line represents the 95% percentile value for a 25 gene window from 1,000 random bootstrap analyses that randomly shuffled the gene's position within the genome for each dataset. All five chromosomes are shown in contiguous order on the *x*-axis with the position labeled using the AGI gene code.

**Figure 4:**
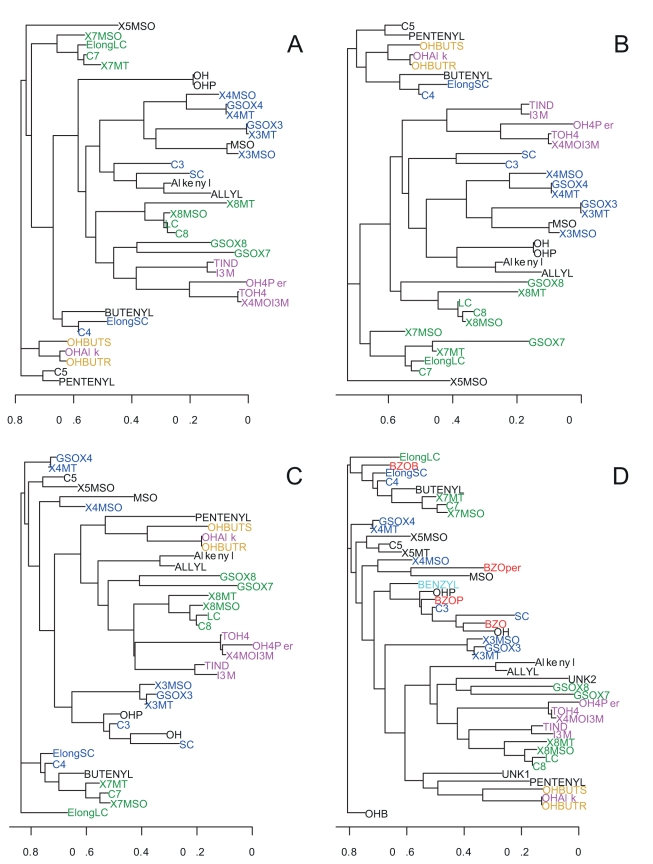
Clustering of traits from control, AgNO_3_-treated leaf, and seedling samples. Shown are neighbor-joining cluster trees of GSL traits where trait-trait distances were estimated based on trait values across the 96 accessions using Spearman's Rank Correlation Coefficient. The GSL were separated into four trait groups and the labels colored based on previous biochemical analysis; INDOLE, indolic GSL (Pink); OHBUT, 2-hydroxy-but-3-enyl GSL traits (Yellow); LC, 7 and 8 C long methionine derived GSL (Green); and SC, 3 and 4 C long methionine derived GSL (Blue). Two Seedling specific trait groups were also included for seedling specific GSL; BZO, benzoyloxy GSL (Red) and Benzyl (Cyan) are phenylalanine derived GSL. Abbreviations are as per Tables S1 and S2. For comparison, only traits available in all three datasets were included in the analysis for (A–C). (A) Relation of GSL traits in the control leaf dataset from the 96 accessions. (B) Relation of GSL traits in the silver leaf dataset from the 96 accessions. (C) Relation of GSL traits from the seedling samples from the 96 accessions. (D) Relation of all GSL traits from the seedling samples from the 96 accessions, including GSL traits not present in mature leaves.

Previous work with untreated GSL leaf samples showed that candidate genes clustered in hotspots, with the two predominant hotspots surrounding the previously cloned *AOP* and *MAM* loci [4], where multiple polymorphisms surrounding the region of these two causal genes significantly associate with multiple GLS phenotypes. We plotted GWA-identified candidate genes for GSL accumulation from the silver and seedling datasets to see if treatment or tissue altered this pattern (Figure 3). Both datasets showed statistically significant (*p*<0.05; Figure 3) hotspots of candidate genes that clustered predominantly around the *AOP* and *MAM* loci with some minor treatment- or tissue-specific hotspots containing fewer genes. This phenomenon is observed across multiple GLS traits (Figure 3). The *AOP* and *MAM* hotspots are known to be generated by local blocks of linkage disequilibrium (LD) wherein a large set of non-causal genes are physically linked with the causal *AOP2/3* and *MAM1/3* genes [4]. Interestingly, while the silver and control leaf GWA datasets showed similar levels of clustering around the *AOP* and *MAM* loci, the hotspot at the *MAM* locus was much more pronounced than the *AOP* locus in the seedling GWA dataset (Figure 3), suggesting more seedling GLS traits are associated with the MAM locus. This agrees with QTL-mapping results in structured RIL populations of *A. thaliana* that have shown that the *MAM/Elong* locus has stronger effects upon seedling GSL phenotypes in comparison to leaves, whereas the effect of the *AOP* locus is stronger in leaves than seedlings [41],[62]–[63]. In addition, the relationship of GSL phenotypes across accessions is highly similar in the two leaf datasets, while the phenotypic relationships across accessions are shifted when comparing the seedling to the leaf (Figure 4). Together, this suggests greater similarity in the genetic variation affecting GSL phenotypic variation between the two leaf datasets than between leaf and seedling datasets, suggesting that GSL variation is impacted more by development than simulated pathogen attack. This is further supported by the analysis of variance (Figure 2).

**Figure 5:**
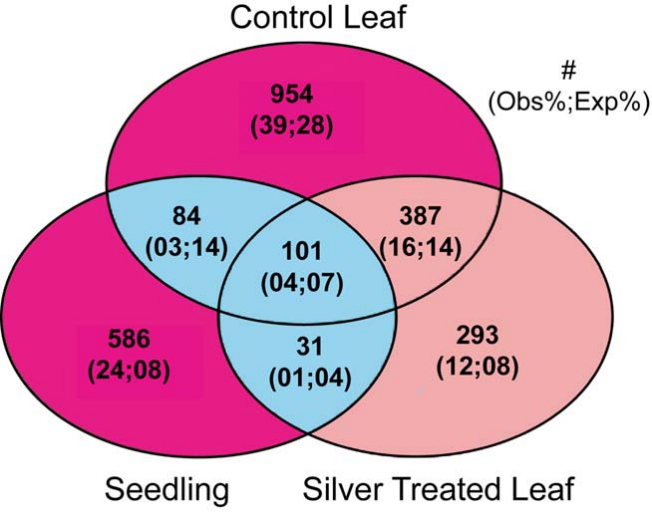
Overlap of significant GWA genes between datasets. A VENN diagram showing the number of GWA significant genes for each dataset: Silver, Control Leaf and Seedling. For this analysis, the genes surrounding the AOP and Elong loci were excluded based upon their previously observed high false-positive rate. The pairs of values in parentheses correspond to the observed and expected percent of total significant genes. In all regions, observed values significantly deviated from expectation (χ^2^ *p*<0.001). Those regions where the observed fraction is less than expected are shown in blue, while regions with more observed significant genes than expected are shown in red.

To further test if measuring the same phenotypes in different tissues or treatments will identify similar GWA mapping candidates, we investigated the overlap of GWA candidate genes identified across the three datasets. For this analysis we excluded genes within the known *AOP* and *MAM* LD blocks as previous research has shown that all of these genes except the *AOP* and *MAM* genes are likely false-positives and would bias our overlap analysis [4],[69]–[71]. The remaining GWA mapping candidate genes showed more overlap between the two leaf datasets than between leaf and seedling datasets (Figure 5). Interestingly, the overlap between GWA-identified candidate gene sets from seedling and leaf data was smaller than would be expected by chance (χ^2^ *p*<0.001 for all three sectors) (Figure 5). This suggests that outside of the *AOP* and *MAM* loci, distinct sets of genetic variants may contribute to the observed phenotypic diversity in GSL across these tissues, which agrees with QTL-mapping studies identifying distinct GSL QTL for seedling and leaf [41],[62]–[63]. As such, focusing simply on GWA mapping candidates independently identified in multiple treatments or tissues to call true significant associations will overlook genes whose genotype-to-phenotype association is conditional upon differences in the experiments. Similarly, the amount of phenotypic variance explained by the candidates differed between the datasets, with control and treated having the highest average explained variance, 39% and 41%, respectively. In contrast, the seedling dataset had the lowest explained variance at 32%, similarly suggesting that altering the conditions of the experiments will change commonly reported summary variables such as explained variance.

### Candidate Gene Network Filtering

GWA studies generally produce large lists of candidate genes, presumed to contain a significant fraction of false-positive associations. One proposed strategy refines these results by searching for enrichment of candidate genes within pre-defined proteomic or transcriptomic networks [15]. To test the applicability of this approach to our GWA study, we overlaid our list of 2,436 candidate genes (excluding genes showing proximal LD to the causal *AOP2/3* and *MAM1/2/3* genes [4]) that associated with at least one GSL phenotype in at least one of the three datasets (Figure 5) onto a previously published co-expression network [72].

If the network filtering approach is valid and there are true causal genes within the candidate gene lists, then the candidate genes should show tighter network linkages to previously validated causal genes than the average gene. Measuring the distances between all candidate genes to all known GSL causal genes within the co-expression network showed that, for all datasets, the GWA candidate genes were on average closer to known causal genes than non-candidates (Figure S4). Interestingly, the GWA mapping candidate genes actually showed closer linkages to the cysteine, homocysteine, and glutathione biosynthetic pathways than to the core GSL biosynthetic pathways, suggesting that natural variation in these pathways may impact *A. thaliana* secondary metabolism (Figure S4 and Dataset S1). The network proximity of GWA mapping candidates to known causal genes supports the utility of the network filtering approach in identifying true causal genes among the long list of GWA mapping candidate genes.

### Candidate Gene Network Filtering (Core Pathway Linkages)

**Figure 6:**
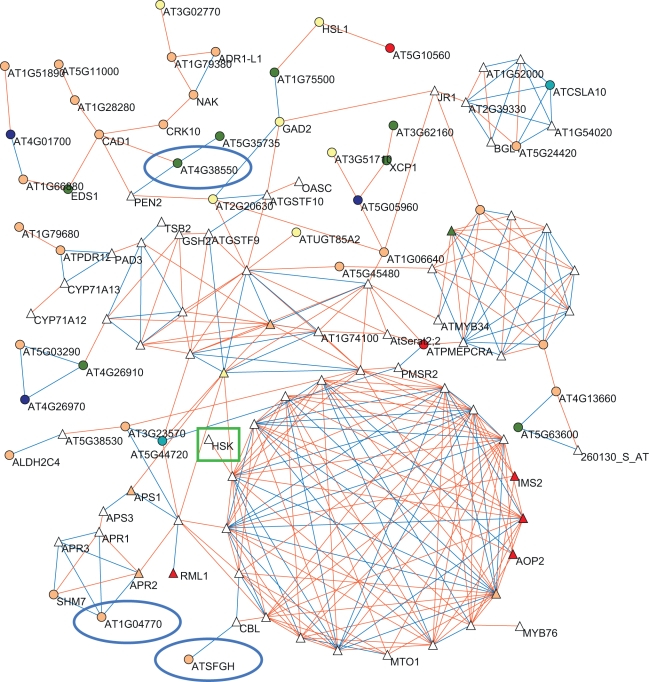
Largest co-expression network of GWA candidates and known GSL core genes. The largest co-expression network involving all genes showing a significant association with a glucosinolate phenotype and the core network of known or predicted glucosinolate genes (Table S7) is shown. Labels for genes with more than five neighbors are not shown due to the density of network. The GSL core is represented elsewhere in a magnified view to provide legibility (Figure S3). Triangles show genes known or predicted to be involved in glucosinolate biology, while circles represent significant GWA candidates not previously linked with glucosinolate production. White symbols show genes with no significant GWA hit in these studies. Other colors show a GWA candidate in the listed dataset: apricot for control; teal for silver; yellow for seedling; olivegreen for control and silver; blue for control and seed; cyan for control and seed; red is for all three datasets. Highlighted are genes mentioned in the text: a green rhombus indicates previously known/predicted GSL-related genes, while blue circles indicate GWA candidate genes that were selected for validation in the current study (Table S9).

To determine if this network filtering approach finds whole co-expression networks or isolated genes, we extended the co-expression network to include known and predicted GSL causal genes (Table S7). The largest network obtained from this analysis centered on the core-biosynthetic genes for the aliphatic and tryptophan derived GSL as well as sulfur metabolism genes (Figures 6 and S3). Interestingly, this large network linked to a defense signaling network represented by *CAD1*, *PEN2*, and *EDS1* (Figure 6) [73].

The defense signaling pathway associated with *PEN2* and, more recently, *CAD2* and *EDS1* had previously been linked to altered GSL accumulation via both signaling and biosynthetic roles [36],[39],[74]–[75]. However, the current network analysis has identified new candidate participants in this network altering GSL accumulation. To test these predicted linkages, we obtained a mutant line possessing a T-DNA insertional disruption of the previously undescribed locus *At4g38550*, which is linked to both *CAD1* and *PEN2* (Figure 6, Table S9). This mutant had elevated levels of all aliphatic GSL within the rosette leaves as well as 4-methoxyindol-3-ylmethyl GSL, shown to mediate non-host resistance (Table S9) [36],[39]. These results suggest a role for *At4g38550* in either defense responses or GSL accumulation.

Network analysis also identified several previously described (*RML1*) and novel candidate (*ATSFGH*, *At1g06640*, and *At1g04770*) genes that were associated with the core-biosynthetic part of the network. *RML1* (synonymous with *PAD2*, *CAD2*), a biosynthetic enzyme for glutathione, has previously been shown to control GSL accumulation either via a signaling role or actual biosynthesis of glutathione [74]–[75]. To test if *ATSFGH* (S-formylglutathione hydrolase, *At2g41530*), *At1g06640* (unknown 2-oxoacid dependent dioxygenase – 2-ODD), or *At1g04770* (tetratricopeptide containing protein) may play a role in GSL accumulation, we obtained insertional mutants. This showed that the disruption of *At1g06640* led to significantly increased accumulation of the short-chain methylsulfinyl GSL but not the corresponding methylthio or long-chain GSL (Table S9). In contrast, the *AtSFGH* mutant had elevated levels of all short-chain GSL along with a decreased accumulation of the long-chain 8-MTO GSL (Table S9). The *At1g04770* mutant showed no altered GSL levels other than a significantly decreased accumulation of 8-MTO GSL (Table S9). This suggests that these genes alter GSL accumulation, although the specific molecular mechanism remains to be identified.

Interestingly, network membership is not sufficient to predict a GSL impact, as T-DNA disruption of homoserine kinase (*At2g17265*), a gene co-expressed with the GSL core but not a candidate from the GWA analysis, had no detectable impact upon GSL accumulation (Table S9).

Thus, the network filtering approach identified genes closely linked to the GSL biosynthetic network that can control GSL accumulation and are GWA-identified candidate genes.

### Candidate Gene Network Filtering (Novel Networks)

**Figure 7:**
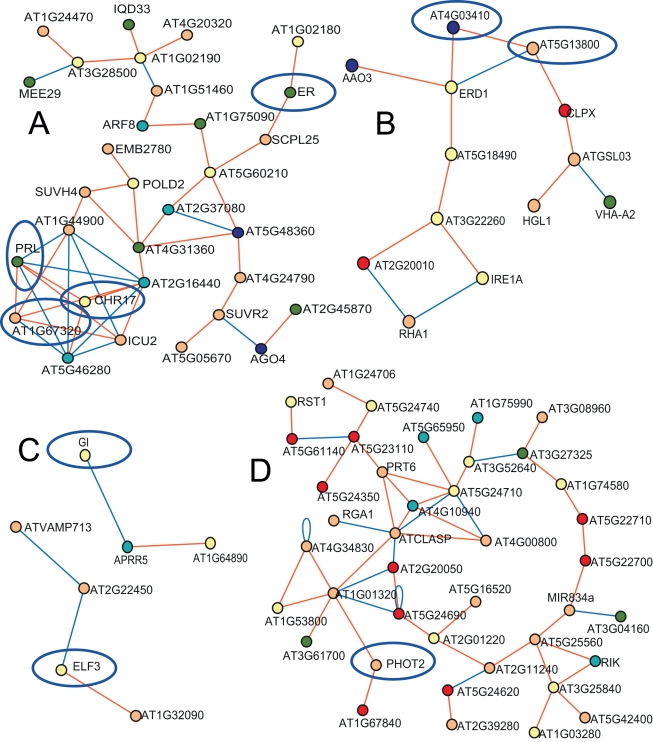
Self-affiliated expression networks of GWA mapping significant candidates. Shown are the co-expression networks that did not involve any GSL-affiliated genes. These networks contain the *ER* locus (A), the *CLPX* locus (B), the *ELF3* and *GI* loci (C), and the *PHOT2* locus (D). Triangles show genes known or predicted to be involved in glucosinolate biology, while circles are other genes. White symbols show those genes with no significant GWA in these studies. Other colors show GWA candidates in the listed dataset: Apricot for control; teal for silver; yellow for seed; olivegreen for control and silver; blue for control and seed; cyan for control and seed; red is for all three datasets. Circled in blue are genes that were selected for validation in the current study (Table S9).

The above analysis shows that GWA candidate genes which co-express with known GSL genes are likely to influence GSL accumulation. However, networks might influence GSL accumulation independent of co-expression with known GSL genes. To test this, we investigated several co-expression networks that involved solely GWA-identified candidate genes and genes not previously implicated in influencing GSL accumulation (Figure 7). Three of these networks included genes that affect natural variation in non-GSL phenotypes within *A. thaliana*, namely *PHOTOTROPIN* 2 (*PHOT2*), Erecta (*ER*) [76], and *ELF3/GI* (Figure 7) [77],[78]. The fourth network did not involve any genes previously linked to natural variation (Figure 7). We obtained *A. thaliana* seed stocks with mutations in a subset of genes for each of these three networks to test whether loss of function at these loci affects GSL accumulation.

The largest network containing no previously known GSL-related genes that we examined is a blue light/giberellin signaling pathway represented by *PHOT2* (Figure 7A). This pathway had not been previously ascribed any role in GSL accumulation in *A. thaliana*. We tested this GWA-identified association by measuring GSL in the single and double *PHOT1/PHOT2* mutants [79]. *PHOT1* was included as it has been shown to function either redundantly or epistatically with *PHOT2* [79]. The single *phot1* or *phot2* mutation had no significant effect upon GSL accumulation (Table S9). The double *phot1/phot2* knockout plants showed a significant increase in the production of detected methylthio GSL as well as a decrease in the accumulation of 3-carbon GSL compared to control plants. Thus, it appears that GSL are influenced by the *PHOT1/PHOT2* signaling pathway, possibly in response to blue light signaling (Table S9). This agrees with previous reports from *Raphanus sativa* that blue light controls GSL [80],[81].

The second non-GSL network we examined contains the *ER* gene (Figure 7B). The ER (*Erecta*) network and specifically the ER locus had previously been queried for the ability to alter GSL accumulation using two Arabidopsis RIL populations (L*er*×Col-0 and L*er*×Cvi) that segregate for a loss-of-function allele at the *ER* locus [41],[51],[63],[82]–[86]. In these analyses, the *ER* locus was linked to seed/seedling GSL accumulation in only one of the two populations and not linked to mature leaf GSL accumulation [41],[86]. Analysis of the *ER* mutant within the Col-0 genotype showed that the *Erecta* gene does influence GSL content within leaves as suggested by the GWA results (Table S9, Figure 7A). Plants with loss of function at *Erecta* showed increased levels of methylthio GSL, long-chain GSL, and 4-substituted indole GSL (Table S9). Interestingly, the ER network contains a number of chromatin remodeling genes. We obtained *A. thaliana* lines with loss-of-function mutations in three of these genes (Table S9) to test if the extended network also alters GSL accumulation. Mutation of two of the three genes (*At5g18620* – *CHR17* and *At4g02060* – *PRL*) was associated with increased levels of short-chain aliphatic GSL and a corresponding decrease in long-chain aliphatic GSL (Table S9). This shows that the *Erecta* network has the capacity to influence GSL accumulation.

Two smaller networks containing the *ELF3* and *GI* genes were of interest as these two genes are associated with natural variation in the *A. thaliana* circadian clock (Figure 7C) [77],[87],[88]. GSL analysis showed that both the *elf3* and *gi* mutants had lower levels of aliphatic GSL than controls (Table S9). Comparing multiple *gi* mutants from both the Col-0 and L*er* genetic backgrounds showed that only *gi* mutants in the Col-0 background altered GSL accumulation (Table S9). This suggests that *gi*'s link to glucosinolates is epistatic to other naturally variable loci within the genome, as previously noted for natural *GI* alleles in relation to other phenotypes (Table S9) [78]. An analysis of the *elf4* mutant which has morphological similarities to *elf3-1* but was not a GWA-identified candidate showed that this mutation did not alter GSL accumulation. Thus, *elf3/gi* affects GSL via a more direct mechanism than altering plant morphology. Given two genes in the circadian clock network directly affects GSL accumulation and given the expression of these two genes are correlated with other genes in the network, it is fair to hypothesize that circadian clock plays a role in GSL accumulation.

While the GSL phenotypes of the above laboratory-generated mutants suggest that variation in circadian clock plays a role in GSL accumulation, they do not prove that the natural alleles at these genes affect GSL accumulation. To validate this, we leveraged germplasm developed in the course of previous research showing that natural variation at the *ELF3* locus controls numerous phenotypes, including circadian clock periodicity and flowering time [77]. We utilized quantitative complementation lines to test if natural variation at *ELF3* also generates differences in GSL content [77]. This showed that the *ELF3* allele from the Bay-0 accession was associated with a higher level of short chain aliphatic GSL accumulation in comparison to plants containing the Sha allele (Table S9). In contrast, both Bay-0 and Sha allele-bearing plants had elevated levels of 8-MTO GSL in comparison to Col-0 (Tables S8 and S9). Thus, *ELF3* is a polymorphic locus that contains multiple distinct alleles that influence GSL content within the plant and the *ELF3/GI* network causes natural variation in GSL content.

The final network examined here, represented by *CLPX* (CLP protease), is likely involved in chlorophyll catabolism and possibly also chloroplast senescence [89]. This network is uncharacterized and has not previously been associated with GSL accumulation or natural variation in any phenotype, but participation in chloroplast degradation is suggested by transcriptional correlation of *CLPX* with several catabolism genes. Analysis of mutants deficient in function for two of these genes showed that they all possessed increased aliphatic GSL in comparison to wild-type controls. These results suggest that natural variation in this putative network could influence GSL content in *A. thaliana*. The majority (12 of 13) of genes in this network show significant variation in transcript abundance across *A. thaliana* accessions, a significantly greater proportion than expected by chance (X^2^ *p*<0.001) [90]–[92], further suggesting that this network may contribute to GSL variation across the accessions.

Finally, we tested a single two gene network found in the co-expression data wherein both genes had been annotated but not previously linked to GSL content. This network involved *AtPTR3* (a putative peptide transporter, *At5g46050*) and *DPL1* (a dihydrosphingosine lyase, *At1g27980*). T-DNA mutants in both genes appeared to be lethal as we could not identify homozygous progeny. However, comparison of the heterozygous progeny to wildtype homozygotes showed that mutants in both genes led to elevated levels of aliphatic GSL (Table S9). Thus, there are likely more networks that are causal for GSL variation within this dataset that remain to be tested.

### Negative Network T-DNA Test

While GSL are considered “secondary” metabolites, these compounds are affected by many aspects of plant metabolism, thus GSL phenotyping is sensitive to any genetic perturbation that affects plant physiology. As such, we identified six genes that were expressed in mature leaves but did not show any significant association of DNA sequence polymorphism with GSL phenotypes and were additionally not identified within any of the above co-expression networks. Insertional mutants disrupted at these loci were designated as random mutant controls (Table S9). Analyzing GSL within these six lines showed that on average 13%±4% of the GSL were affected in the random control mutant set even after correction for multiple testing. While this suggests that GSL may be generally sensitive to mutations affecting genes expressed within the leaf, this incidence of significant GSL effects is much lower than observed for the T-DNA mutants selected to test GWA mapping-identified pathways (CLPX - 78%±11%, PTR3 – 61%±6%, Erecta – 45%±10%, GSL – 46%±11%, ELF3/GI – 53%±17%). In all cases the mutants deficient in GWA pathway-identified gene function showed significantly greater numbers of altered GSL phenotypes than the negative control T-DNA mutant set (X^2^, *p*<0.001), suggesting that combining GWA-identified candidate genes with co-expression networks successfully identifies genes with the capacity to cause natural variation in GSL content. Identifying the specific mechanisms involved will require significant future research.

## Discussion

The influence of conditional genetics, i.e. interaction of genotypes with environment or development, has been intensively studied within structured mapping populations and shown to exert considerable influence on the accumulation of small metabolites [20],[49]–[51],[93]–[94]. However, conditional effects have not been routinely included in GWA studies. In this report, we show extensive variation in the identification of GWA candidate genes that depends upon both Genotype×Environment and Genotype×Tissue interactions. The analysis of GSL accumulation in two different tissues showed a significant bias toward indentifying different causal genes for the GSL phenotypes in the two different tissues (Figure 5). As such, conditional genetics are likely to be as critical in GWA analyses as for QTL analyses using structured populations. This suggests that requiring replication of genotype-phenotype associations across environments or conditions as a condition for validation, as has been suggested for human GWA studies, may lead to a significant bias against loci that interact with the environment or development. Instead, methods should be developed to specifically target these loci.

Interestingly, developmental differences played a larger role than the AgNO_3_ treatment in influencing genetic variation across this collection of accessions, as displayed by the distribution of phenotypes and their variance across the datasets (Figures 2 and 3). The different developmental stages, seedling and mature leaf, showed a non-random distribution of GWA candidate genes with repulsion, such that a seedling candidate was less likely to be a leaf candidate gene than would be expected by random chance. This result has two implications. The first is that GSL are influenced by different genetic variation in the different developmental stages. This is not unexpected given the changing herbivore pressures that the plant will encounter over the course of its development. Production of different optimal GLS profiles for defense at each developmental stage likely is mediated by different genetic networks. The second implication is that a large number of genes may have the potential to influence GSL accumulation.

### Network Proximity as a Method to Filter GWA Candidates

A limiting factor for the utility of GWA studies has been the preponderance of false-positive and false-negative associations which makes the accurate prediction of biologically valid genotype-phenotype associations very difficult. In this report, we describe the implementation and validation of a candidate gene co-expression filter that has given us a high success rate in candidate gene validation (>75%). The co-expression dataset is derived from transcript accumulation within a single *A. thaliana* accession (Col-0) across a wide range of developmental and environmental states [72]. This dataset has previously been used to show that genes showing co-expression often modulate the same phenotype, and may thus also function within the same pathway [57]–[59],[95]–[99]. This co-expression dataset provides a functional grouping of *A. thaliana* genes based upon non-genetic variation. This provides an orthogonal grouping to that provided by the GWA mapping which associates genes to phenotypes via natural genetic variation. This approach is similar to other filtering approaches that utilize complementary datasets to rank candidate genes [11],[100]–[102]. However, most of these other approaches utilize two databases, e.g. GWA and eQTL (expression quantitative trait loci), that are both based upon natural genetic variation and thus do not provide independent filters [11],[100]–[101]. In contrast to these other network approaches, our methodology does not rely upon a statistical rank or enrichment procedure which can be dominated by individual genes with high significance possibly due to GWA mapping artifacts [102]. Instead, our approach focuses upon relative network size to direct the researcher to the most interesting candidate networks. This approach is less susceptible to statistical artifacts and allows the user to input bait genes suggested by a priori knowledge [95],[103]–[104]. This approach should be useful in any system possessing genomic networks that are orthogonal to the GWA-identified candidate gene lists.

### Number of Genes Determining a Phenotype's Level and Proximity of Effect

The use of multiple tissues and treatment conditions, as well as a large set of different but related GSL phenotypes, led to the identification of several thousand candidate genes. Even after decreasing this number by using the network expression filter approach, several hundred candidate genes of interest remained. Analysis of a set of these genes via plants bearing single gene mutations showed that disruption of many of these genes can alter the amount or pattern of GSL accumulation (Table S9 and Figures 6 and 7). Given the observation that the background genotype can influence the capacity to identify a mutational effect (see *gi* mutants in L*er* v Col-0, Table S9), our estimate of tested genes influencing GSL accumulation is conservative. Given this, it is likely that a very large number of small to moderate effect loci influence GSL accumulation within *A. thaliana*, echoing recent findings regarding the genetics of human height, and maize flowering time [105]–[106]. This suggests that the whole genome may have a pattern similar to that found in an analysis of a single Arabidopsis locus that identified several QTL for growth within a small section of the genome [70]. As such, it might be common for quantitative traits to be influenced by thousands of causal loci [107].

The potential existence of thousands of polymorphic genes influencing a phenotype raises a common concern that these effects actually represent indirect pleiotropy, where moderate to small effects of a locus upon a phenotype are not biologically significant and do not reflect direct molecular control of the trait. However, numerous studies on GSL variation within wild populations have shown that changes in GSL accumulation similar to those identified here have selective consequences in field studies [33]–[35],[43]–[45],[108]. As such, even if polymorphisms in these identified genes have indirect pleiotropic effects upon GSL accumulation, these changes have a strong potential to influence *A. thaliana* in natural settings. Thus, it may be more useful to consider, instead of indirect versus direct effects of a locus, a continuous distribution that describes the number of molecular steps required to link a particular gene to the most proximal controller of the phenotype—in this case, an enzyme in the biosynthetic pathway. This raises the distinct problem of adaptive constraint wherein natural variation at a locus is limited by its indirect consequences upon other phenotypes. For instance, a *phototropin* allele with a beneficial effect on seedling phototropic behavior may be limited in its selective advantage due to a deleterious effect on GSL accumulation [109]–[110]. While this possibility remains to be tested in natural populations, it invites the question of why these phenotypic linkages occur. Is there a benefit to the influence of these loci on GSL accumulation, or has insufficient time passed since the de novo evolution of GSL biosynthesis to generate the genetic modularity to bypass historical linkages between development and metabolism [111]?

### Number of Genes Influencing a Phenotype and Validation Barriers

A more mundane but significant experimental challenge of generating a list of thousands of candidate genes potentially causing natural variation in a phenotype is validation. Even after our expression network filtering, we were left with hundreds of likely candidates that would take decades to rigorously validate. Given that it is likely that at least several hundred genes lead to natural variation in GSL accumulation [105]–[106], how do we validate the effects of natural alleles at these loci, and is it worth the effort? If it is not worth the effort for GSL accumulation, what deciding factors should determine when a single phenotype should be completely dissected (to the level of knowing all genes containing a causal link to natural variation within a phenotype)? Given the importance of quantitative variation in numerous agronomic and medically important phenotypes, this discussion needs to begin, because untested presumptions about the number of causal genes for a phenotype greatly influences current GWA research and associated strategies for avoiding false-positive and false-negative results [2],[65],[112].

### GWA and Development

We identified significant differences in GSL accumulation between two different developmental stages and this led to the identification of GWA candidate genes. While previous work on structured mapping populations, such as RILs, has shown that each tissue may be viewed as a distinct genetic module for both development and biochemistry [41],[49]–[50],[113]–[114], this is one of the first reports about tissue differences in an unstructured population. This tissue specificity indicates that it is not possible to simply require a candidate gene to replicate across tissues to validate its GWA signature. Instead, each tissue has to be looked at as a potentially independent modular system [115]. Such modularity could be mediated by members of a gene family each acting in a limited set of tissues, either as a result of sub- or neo-functionalization [116]–[119]. Both sub- and neo-functionalization have played an important role in the evolution of GSL and other plant secondary metabolites [55],[69],[92],[96]. The impact of development on GWA remains to be tested across a broader range of tissues and developmental stages.

### Conclusion

In this report, we show that GWA-mapping, like QTL-mapping using structured populations, is sensitive to interaction of genetic variation with the environment and the developmental stage of phenotype measurement. This has not often been considered as a critical factor influencing GWA studies, given the difficulty of obtaining replicated analyses within organisms such as humans. Future work incorporating systematic analysis of how GWA studies are influenced by developmental or environmental gradients will be critical to understanding how the genomic architecture of a species controls its phenotypes. We have developed and validated a new approach to identifying GWA candidate genes and shown that the use of orthogonal genomic network datasets can lead to a very high success rate in the biological validation of candidate genes. This new approach, in combination with the observation of conditional GWA results, suggests that large numbers of genes can have a causal connection to variation within GSL and other phenotypes.

## Materials and Methods

### Population, Treatment, and Growth Conditions

A previously described collection of 96 natural *A. thaliana* accessions was used to measure GSL accumulation for GWA mapping with existing SNP data from these same lines [3],[27]–[28],[120]. Seeds were imbibed and cold stratified at 4°C for 3 d to break dormancy. Seeds were planted in a randomized block design, with multiple seeds of each accession occupying an individual cell within 36-cell flats (approximately 100 cm^3^ soil volume per cell). Four plantings of the 96 accessions provided four independent replicates for each accession. At 1 wk of age, seedlings were thinned to leave one plant per cell and glucosinolates were extracted from 10 of the removed seedlings. For all experiments, plants were maintained under short day conditions in controlled environment growth chambers. At 35 d post-germination, two fully expanded mature leaves were harvested, digitally photographed, and one was directly analyzed for GSL content as described below [18],[121]. The other leaf was treated with 5 mM AgNO_3_ for 48 h prior to harvest for GSL analysis. AgNO_3_ induces plant responses to pathogens by interfering with ethylene hormone-signaling and inducing reactive oxygen species. We utilized AgNO_3_ as a treatment to estimate the effect of variation in plant defense response upon GWA mapping [122]–[124]. In total, these datasets contain four measurements per accession per tissue and treatment for a total of 301 assays of seedling GSL (Seedling Dataset), 374 assays of control leaf GSL (Ctl Dataset), and 375 assays of GSL following AgNO_3_ treatment of leaves (Silver Dataset). The data for the control dataset is reported elsewhere as the “2008 dataset” [4].

### Analysis of GSL Content

GSL content of excised leaves and seedlings was measured using a previously described high-throughput analytical system [62],[69]. Briefly, for excised leaves, one leaf was removed from each plant, photographed, and placed in a 96-well microtiter plate with 500 µL of 90% methanol and one 3.8 mm stainless steel ball-bearing. Seedlings were removed from pots with forceps, gently cleaned with distilled water to remove soil, and similarly placed into 90% methanol in microtiter plates. Tissues were homogenized for 2 min in a paint shaker, centrifuged, and the supernatants transferred to a 96-well filter plate with 50 µL of DEAE sephadex. The sephadex-bound GSL were eluted by overnight, room temperature incubation with sulfatase. Individual desulfo-GSL within each sample was separated and detected by HPLC-DAD, identified, and quantified by comparison to purified standards [125]. Tissue area for each leaf was digitally measured using Image J with scale objects included in each digital image [126]. The GSL traits are reported per cm^2^ of leaf area for the mature leave data or per seedling for the seedling data. There was no significant variation detected for leaf density within these accessions (unpublished data). In addition to the content of individual GSL, we developed a series of summation and ratio traits based on prior knowledge of the GSL pathways [127]. These ratios and summation traits allow us to isolate the effects of variation at individual steps of GSL biosynthesis from variation affecting the rest of the biosynthetic pathway [127].

### Parititioning H^2^ Between Structure and Accession

To estimate broad-sense heritability due to accession and population structure for the different metabolites, we evaluated the data using a model where the metabolite traits are y_sar_ = *μ*+S_s_+A(S)_sa_+T_t_+R(T)_tr_+T_t_:S_s_+T_t_:A(S)_sa_+ε_sart_ where s = 1,…,8; r = 1,…4; t = 1,2; and a = 1,…,95. The main effects are denoted as S, A, T, and R and represent structure, accession, treatment (or tissue), and replicate block, respectively. Here, the variable T may refer to (1) treatment corresponding to the two factors with or without AgNO_3_ treatment or (2) tissue corresponding to the two factors' mature leaves or seedlings. Population structure is represented as s = 1,…,8, corresponding to eight distinct groups into which these 96 accessions have previously been assigned [27]–[28]. The error, *ε_sart_*, is assumed to be normally distributed with mean 0 and variance σ_ε_^2^. Broad-sense heritability was estimated as the percent of total variance attributable to accession nested within structure and that for structure was estimated as the percent of total variance attributable to structure. The data were analyzed independently for the two treatments or conditions: control versus AgNO_3_ and control versus seedling (Figure 2; Tables S1 and S2).

### Association Mapping

To conduct single-locus GWA mapping accounting for population structure, we adopted a previously published method, the efficient mixed-model association (EMMA) algorithm [65]. EMMA is a statistical mixed model [65] where each SNP is modeled as a fixed effect and population structure, represented as a genetic similarity matrix, is modeled as a random effect. Variance components for this mixed model were estimated directly using maximum likelihood as implemented in the R/EMMA package [65]. Within this model, the independent measures of each metabolite within each accession, obtained from the analysis of variance model y_sar_ = *μ*+A_a_+*R_r_*+*ε_sar_*, were directly incorporated as genetic averages for the accessions (Tables S3 and S4). Because GWA was performed independently for each of the three datasets and because EMMA accounts for population structure, the variables S_s_, T_t_, and R_r_ were excluded in this model. The average GSL accumulation per accession for the control dataset is reported elsewhere as the “2008 experiment” [4]. The full results are available at http://www.plantsciences.ucdavis.edu/kliebenstein/supplementaldataset1.zip.

### Calling Positive Genes for GWA Mapping

We utilized a previously reported criterion for calling significant gene-trait associations in these three datasets [4]. *p* value distributions of the GWA analysis were not uniform. Accepting an inherently elevated false-positive rate, we identified SNP within the bottom 0.1 percentile of each *p* value distribution, corresponding to each trait, as significant for EMMA. Given previous observations that multiple SNPs per gene are typically associated with a trait for true-positives [30], we developed a criterion for calling a significant association between a trait and a gene [4],[30]: requiring at least two significant SNPs within ±1 kb of a gene's coding region to call a gene significant. This approach optimized the ratio of empirical false-positive to false-negative associations. This criterion was independently applied to the GWA results from all tissues and conditions (Tables S5 and S6).

### Estimating Phenotypic Variance Controlled by GWA Candidates

We estimated the variance explained by the candidate GWA mapping genes identified in this study using the GenABEL package in R [66]–[67]. This was done using a mixed polygenic model of inheritance for each phenotype within each dataset. Only SNPs within 1 kb of significant genes were utilized.

### Co-Expression Network Analyses

Co-expression data were obtained from ATTED II [72],[128]. We extracted correlation values for transcript levels of genes showing significant association in at least one of the three datasets (Tables S5 and S6) [4] as well as a list of genes with predicted or known roles in GSL metabolism or regulation (Table S7). This latter set of genes was included to act as “bait genes” that might catalyze network formation around a known causal gene [59],[95],[98]. GWA candidates located within previously identified regions surrounding the *AOP* and *MAM* loci were then excluded to reduce detection of false associations due to linkage with the causal *AOP2/3* and *MAM1/2/3* genes [4]. Co-expression networks were constructed between these genes using a Mutual Rank threshold of up to 15 [129]. Co-expression networks were visualized using Pajek [130].

To test if GWA-identified candidate genes showed tighter linkage to known GSL networks than expected by chance, the shortest paths between each candidate or randomly selected control gene and all verified GSL genes within the full co-expression network were compared using the R/igraph package [67],[131]–[133]. This analysis was performed independently for candidate genes found in the control, silver, or seedling datasets as well as for all GSL genes and a subset of randomly selected genes that were not significantly associated with GSL phenotypes within the GWA mapping (Figure S4). This analysis generated a distribution of path distances linking the set of GWA mapping candidate genes to the known GSL genes. We also repeated the analysis by dividing the GSL genes into each of the specific biosynthetic pathways to test if any specific pathways showed reduced path distances to GWA mapping candidates (Tables S7 and S8) [50],[92],[99],[134]–[135].

We conducted two statistical tests to compare the null distribution (distances from non-significant genes to known GSL genes) with the GWA mapping candidate distribution (distances from GWA candidate genes to known GSL genes). The Wilcoxon Rank Sum Test tests the probability of a location shift between the distribution of the shortest paths of all GWA mapping candidate genes (from one of the three datasets) to all known GSL genes and the distribution of the shortest paths of all non-significantly associated genes to the all known GSL genes. The Ansari-Bradley Test examines the probability that the two aforementioned distributions are differently dispersed. Both statistic tests were conducted using the full GSL network list as well as each individual biosynthetic pathway (Tables S7 and S8).

### GWAS Candidate Gene Selection and Validation

We focused our validation efforts on a set of GWA-identified candidate gene co-expression networks that exhibited different numbers of genes that are a member of the network (levels of membership). Criteria for selection of candidate genes from these networks for testing were connectedness (the gene had to show correlated expression levels (MR rank of <16) with multiple candidate genes within the network) and availability of viable mutants. These mutants were either a pre-existing characterized mutant line or a homozygous T-DNA mutation within an early exon of the candidate gene available from the Arabidopsis Biological Resource Center (ABRC) [136]. For each network tested, we attempted to test at least four separate genes within the network for altered GSL accumulation. We obtained putative homozygous T-DNA mutants for 18 candidate genes and validated their homozygosity using a PCR assay. Primers for the assay were designed using the SALK SIGnAL iSect primer design tool (http://signal.salk.edu/tdnaprimers.2.html). Of the 18 T-DNA mutants surveyed, homozygous mutants could not be obtained for 11 mutants, likely from lethality. In these cases, heterozygote lines were allowed to self-pollinate, and homozygous seed stocks were obtained by single seed decent following PCR-based genotyping of the progeny. In the absence of a homozygous line, we tested GSL content within the adult rosette leaves within PCR-confirmed heterozygous individuals. We also obtained mutants deficient in function at the following loci: *phototropin1/phototropin2* (*phot1/phot2*) (4 lines), Gigantea (*gi*) (8 alleles), Erecta (*er*) in Col-0, and *early flowering 3-1* (*elf3-1*) [79],[137]–[140]. Plants were grown under 10 h of light for 5 wk using a randomized complete block design over two experiments with at least four biological replicates per experiment. Leaf area and GSL content of the first true leaf was obtained as described above. A Dunnett's *t*-test was conducted to test the statistical significance of differences in GSL content between the mutant and wild-type while correcting for multiple comparisons using the R/multcomp package (Table S9) [141]. GSL were measured in at least two biological replicates per genotype, averaging 17 total individual measurements per genotype across the two replicates (min = 8, max = 48) (Table S9). Only wild-type controls grown concurrently with the mutants were used for the statistical comparison.

### Measuring Glucosinolate Accumulation between the Bay-0 and Sha *ELF3* Alleles

We utilized previously generated quantitative complementation lines to validate that natural variation in the *ELF3* locus did alter GSL accumulation [77]. *elf3:Bay-0* and *elf3:Sha* transgenic T1 seeds were planted on soil including *elf3.1* mutants and wild-type Col-0 as a control [77]. The extreme hypocotyl length and cotyledon color phenotypes of the *elf3.1* mutants were assessed to distinguish transformed from untransformed plants [137]. Transformed plants were grown for 25 d in a 10 h photoperiod. At 25 d, leaf tissue was harvested from each plant and individually extracted and assayed via HPLC for glucosinolate composition and concentration as previously described [41],[69]. The experiment was replicated 5 times for a total of 41 *elf3:Bay-0* and 44 *elf3:Sha* independent T1 plants. GSL differences between the two ELF3 alleles were tested as described above.

## Supporting Information

Dataset S1 GWA network candidate results. This dataset contains the GWA network candidate output results in a .net file ready for import into Pajek.
(TXT)

Figure S1 Trait distributions from leaf-control, leaf-AgNO_3_, and seedling datasets. Distributions of total aliphatic (left) and total indolic (right) glucosinolates are shown as examples to illustrate the differences between the three datasets. Seedling glucosinolates are presented in amount per seedling to control for differences in cellular expansion.
(TIF)

Figure S2 VENN Diagram of positive calls and trait groups. VENN diagrams showing the numbers of GWAS significantly associated genes for each dataset, Silver, Control, and Seedling, are shown. The GSL were separated into four trait groups based on previous biochemical analysis; INDOLE, indolic GSL; OHBUT, 2-hydroxy-but-3-enyl GSL traits; LC, 7 and 8 C long methionine derived GSL; SC, 3 and 4 C long methionine derived GSL. Two Seedling specific trait groups were also included for seedling specific GSL; BZO, benzoyloxy GSL and Benzyl are phenylalanine derived GSL. The bottom right VENN diagram displays overlap between the four common trait groups and the seedling specific groups.
(TIF)

Figure S3 Core GSL co-expression network. The known or predicted GSL genes generate a core GSL co-expression network that is expanded in this presentation for legibility. The general biochemical functions of the four major clusters within this super network are labeled. Three of the major clusters are further magnified to provide gene identification.
(PDF)

Figure S4 Distributions of shortest distances between known GSL genes and GWA candidates. Shown are plots comparing the distributions of the shortest distances between known GSL genes and GWA candidates for the control (red), silver (green), and seedling (blue) datasets. For comparison similar distributions derived from non-GWA-candidates (all genes) are also shown (black lines). Pw is the Wilcoxon Rank Sum test *p* value comparing the probability of a location shift between the distribution of the shortest paths of all GWA candidate genes (from one of the three datasets) to the corresponding glucosinolate gene and the distribution of the shortest paths of all non-significantly associated genes to the corresponding glucosinolate gene. Pa is the Ansari-Bradley Test probability assessing the difference in dispersion between the two aforementioned distributions. This was done for all GSL genes as well as for each of the specific biosynthetic networks as defined.
(PDF)

Table S1 Estimates of variance components for GSL in AgNO_3_ study. For each glucosinolate trait the following model was examined: y_sar_∼*μ*+S_s_+A(S)_sa_+Tt+R(T)_tr_+T_t_:S_s_+T_t_:A(S)_sa_, where μ is the intercept, s is the K ∈ {1,…,8) value for the corresponding accession [27]–[28], A(S)_sa_ is the effect of accession nested in structure, T_t_ is the effect of AgNO_3_-treatment, and R(T)_tr_ is the biological/technical replicate of the measure. The model was evaluated by combining the control (untreated mature leaves) and AgNO_3_-treated datasets. F, F-statistic of the model; P(F), nominal *p* value of the F-statistic; DF(num), numerator degrees of freedom; DF(denom), denominator d.f.; R^2^, fraction of total variance explained by the model; η^2^(x), partial R^2^ of the corresponding predictor variable; and P(x), *p* value of the corresponding predictor variable.
(XLS)

Table S2 Estimates of variance components for GSL in seedling study. For each glucosinolate trait the following model was examined: y_sar_∼*μ*+S_s_+A(S)_sa_+Tt+R(T)_tr_+T_t_:S_s_+T_t_:A(S)_sa_, where μ is the intercept, s is the K ∈ {1,…,8) value for the corresponding accession [27]–[28], A(S)_sa_ is the effect of accession nested in structure, T_t_ is the effect of tissue type (mature leaves versus seedlings), and R(T)_tr_ is the biological/technical replicate of the measure. The model was evaluated by combining the control (mature leaves) and seedling datasets. F, F-statistic of the model; P(F), nominal *p* value of the F-statistic; DF(num), numerator degrees of freedom; DF(denom), denominator d.f.; R^2^, fraction of total variance explained by the model; η^2^(x), partial R^2^ of the corresponding predictor variable; and P(x), *p* value of the corresponding predictor variable.
(XLS)

Table S3 Genetic means of glucosinolate abundance per accession for silver treated accessions. All metabolite values are in nmol per mg fresh weight tissue. Shown are the predicted means from four independent plants treated with silver nitrate per accession as per the statistical model: y_sar_ = *μ*+A_a_+*R_r_*+*ε_sar_*. Treated and untreated camalexin values are presented and are considered related to the indole GSL metabolites.
(XLS)

Table S4 Genetic means of glucosinolate abundance per accession for seedlings. All metabolite values are in nmol per seedling. Shown are the predicted means from four independent samples per accession as per the statistical model: y_sar_ = *μ*+A_a_+*R_r_*+*ε_sar_*.
(XLS)

Table S5 Gene-to-trait associations as identified using silver treated samples. Logical table indicating whether each of 31,505 genes is significantly associated to each of the 46 traits within the seedling samples. AGI is the gene code, Chr is the chromosome, and Start and End are the position of the gene in basepairs. For each trait, a gene is significantly associated if at least two SNP within ±1 kb flanking the coding region has a *p* value in the bottom 0.1 percentile of the *p* value distribution. T, is significant; F, not significant and genes with no significances are not listed.
(XLS)

Table S6 Gene-to-trait associations as identified using seedling material. Logical table indicating whether each of 31,505 genes is significantly associated to each of the 46 traits within the seedling samples. AGI is the gene code, Chr is the chromosome, and Start and End are the position of the gene in basepairs. For each trait, a gene is significantly associated if at least two SNP within ±1 kb flanking the coding region has a *p* value in the bottom 0.1 percentile of the *p* value distribution. T, is significant; F, not significant and genes with no significances are not listed.
(XLS)

Table S7 Known and putative genes involved in the GSL pathway. List of genes either known or predicted to play a role in GSL metabolism and regulation. AGI, the AGI (Arabidopsis Genome Initiative) code for each gene; Pathway, specific part of the GSL metabolic system the gene is thought function; Pseudogene, whether or not the gene is predicted to be a pseudogene; Evidence, experimental evidence (Genetic or Biochemical) or sequence evidence base on homology to validated GSL gene (Homology).
(XLS)

Table S8 GSL abbreviations.
(XLS)

Table S9 Mutant analysis for altered GSL accumulation. Chemical and statistical analysis for the various single gene mutants and genotypes queried within the manuscript. The wildtype to mutant comparison being conducted is shown in bold at the start of each subtable. The average value for mutant and control are shown in the top table for each mutant while the standard error is shown in the second table. The *p* value comparing the two genotypes is on the line labeled *p* value and *n* shows the number of independent plants measured per line.
(XLS)

Table S10 Estimated phenotypic variance determined by significant GWAS candidates. Abbreviations per glucosinolate are as described in Table S8. Percent phenotypic variations are as described in Materials and Methods. Analysis was conducted independently for each dataset.
(XLS)

## Abbreviations

EMMA: efficient mixed-model association
eQTLs: expression quantitative trait loci
GSL: glucosinolate
GWA: genome-wide association
LD: linkage disequilibrium
QTL: quantitative trait loci
RIL: recombinant inbred line
