# Supplementary figures and images for: Combining Genome-Wide Association Mapping and Transcriptional Networks to Identify Novel Genes Controlling Glucosinolates in Arabidopsis thaliana
Source: PLoS Biol. 2011 Aug 16;9(8):e1001125. doi: 10.1371/journal.pbio.1001125 (PMC3156686; doi:10.1371/journal.pbio.1001125)

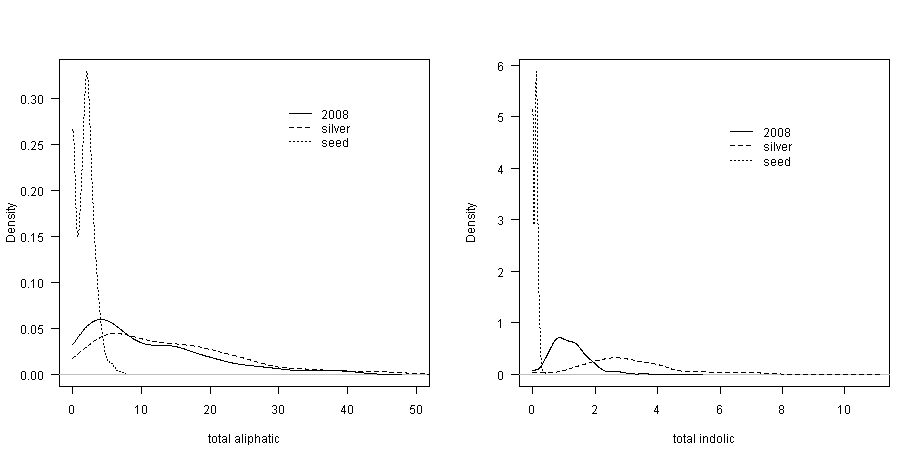

Supplement: Figure S1 — Trait distributions from leaf-control, leaf-AgNO3, and seedling datasets. Distributions of total aliphatic (left) and total indolic (right) glucosinolates are shown as examples to illustrate the differences between the three datasets. Seedling glucosinolates are presented in amount per seedling to control for differences in cellular expansion. (TIF) [file pbio.1001125.s002.tif]

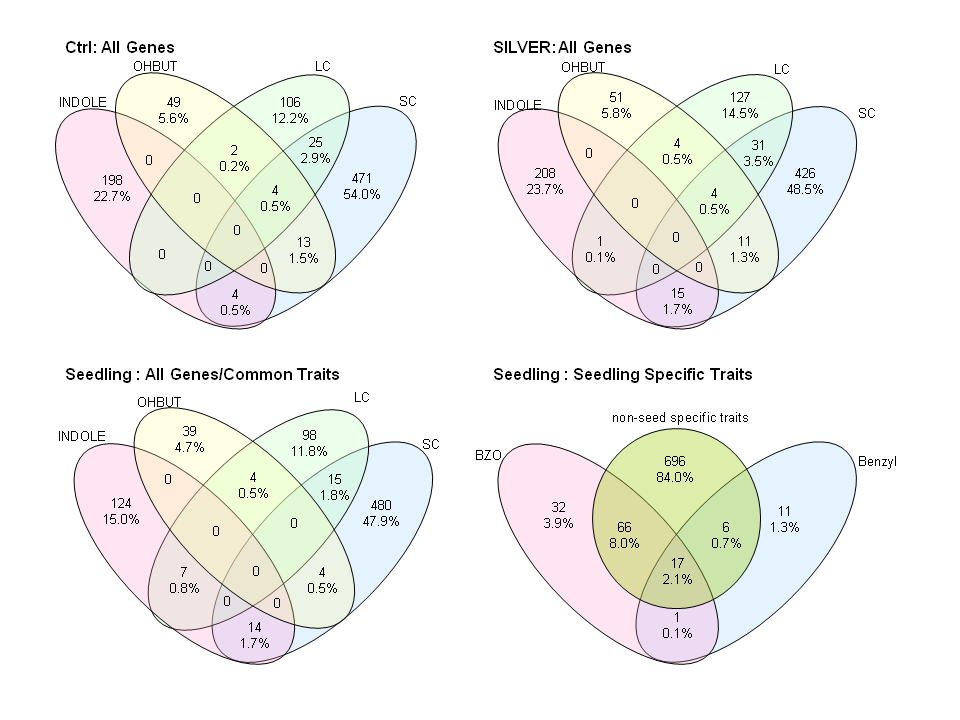

Supplement: Figure S2 — VENN Diagram of positive calls and trait groups. VENN diagrams showing the numbers of GWAS significantly associated genes for each dataset, Silver, Control, and Seedling, are shown. The GSL were separated into four trait groups based on previous biochemical analysis; INDOLE, indolic GSL; OHBUT, 2-hydroxy-but-3-enyl GSL traits; LC, 7 and 8 C long methionine derived GSL; SC, 3 and 4 C long methionine derived GSL. Two Seedling specific trait groups were also included for seedling specific GSL; BZO, benzoyloxy GSL and Benzyl are phenylalanine derived GSL. The bottom right VENN diagram displays overlap between the four common trait groups and the seedling specific groups. (TIF) [file pbio.1001125.s003.tif]

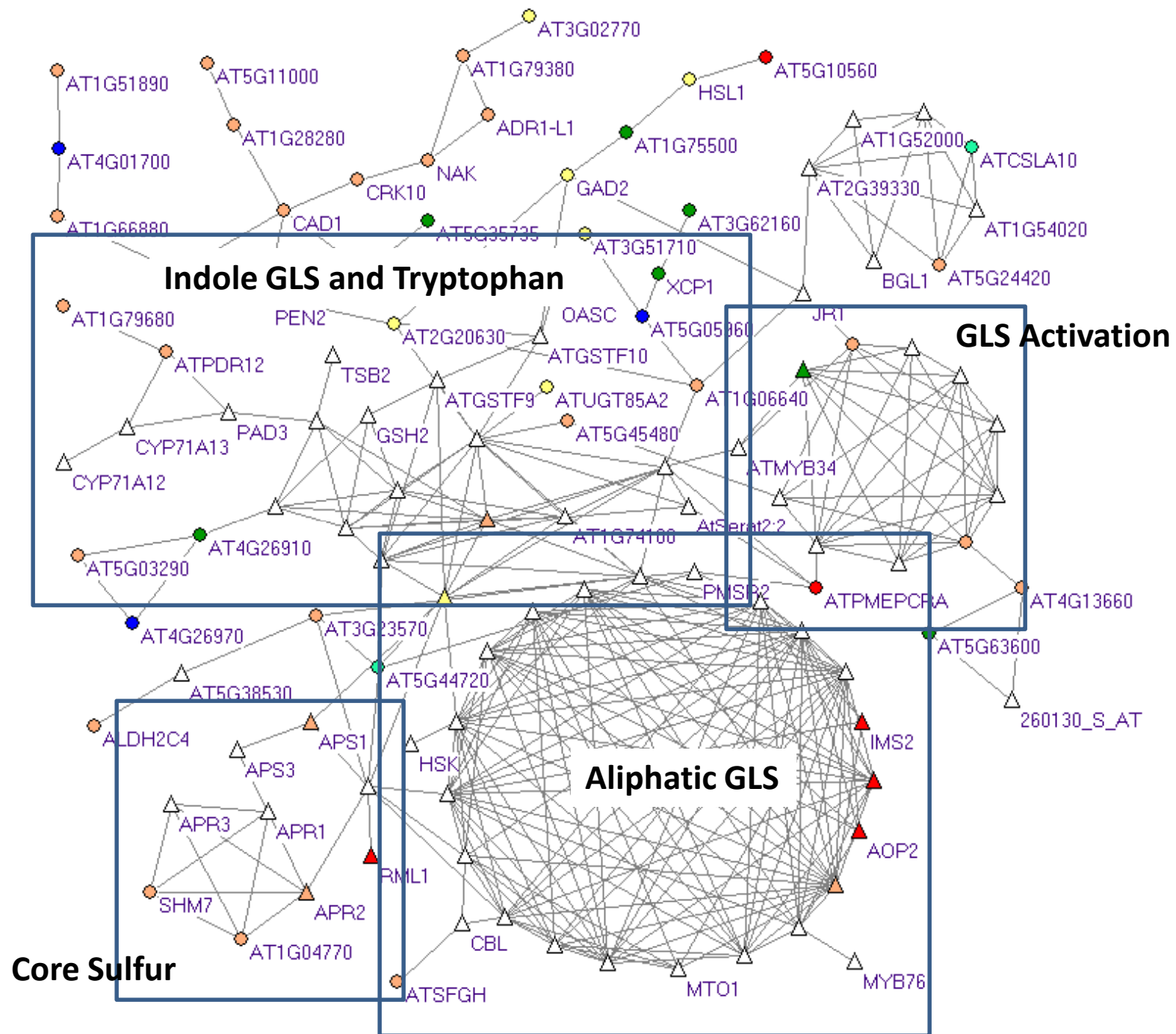

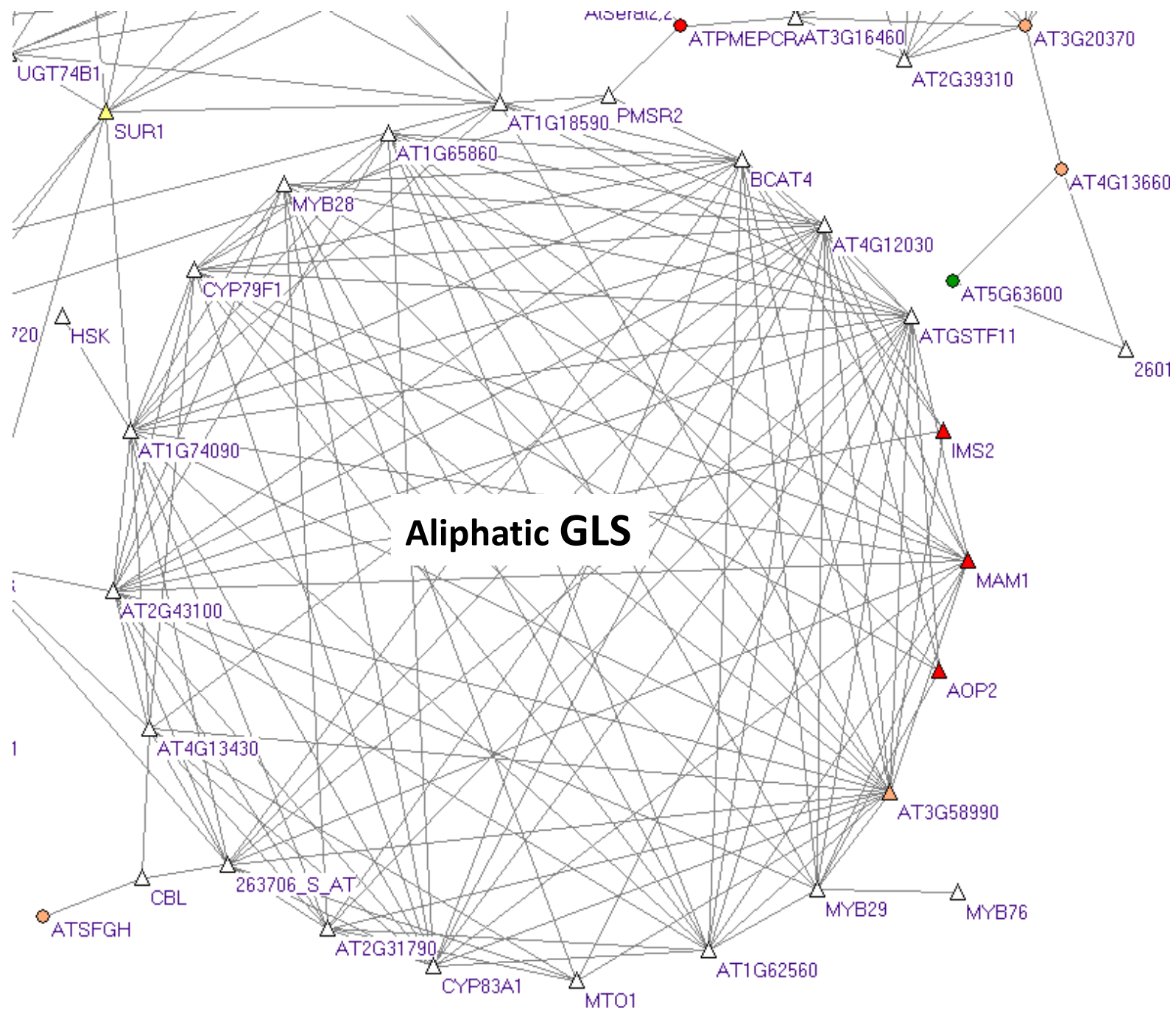

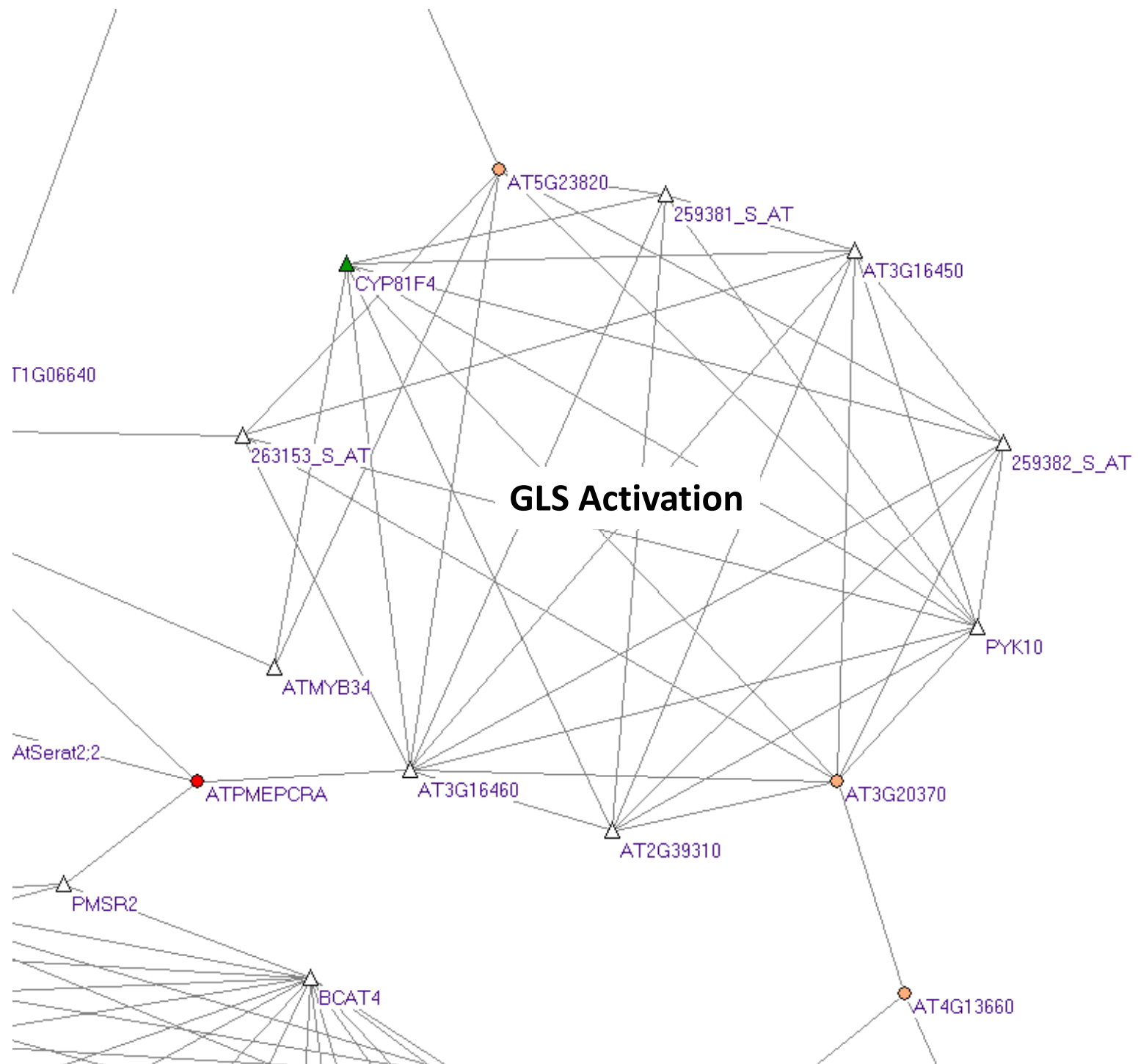

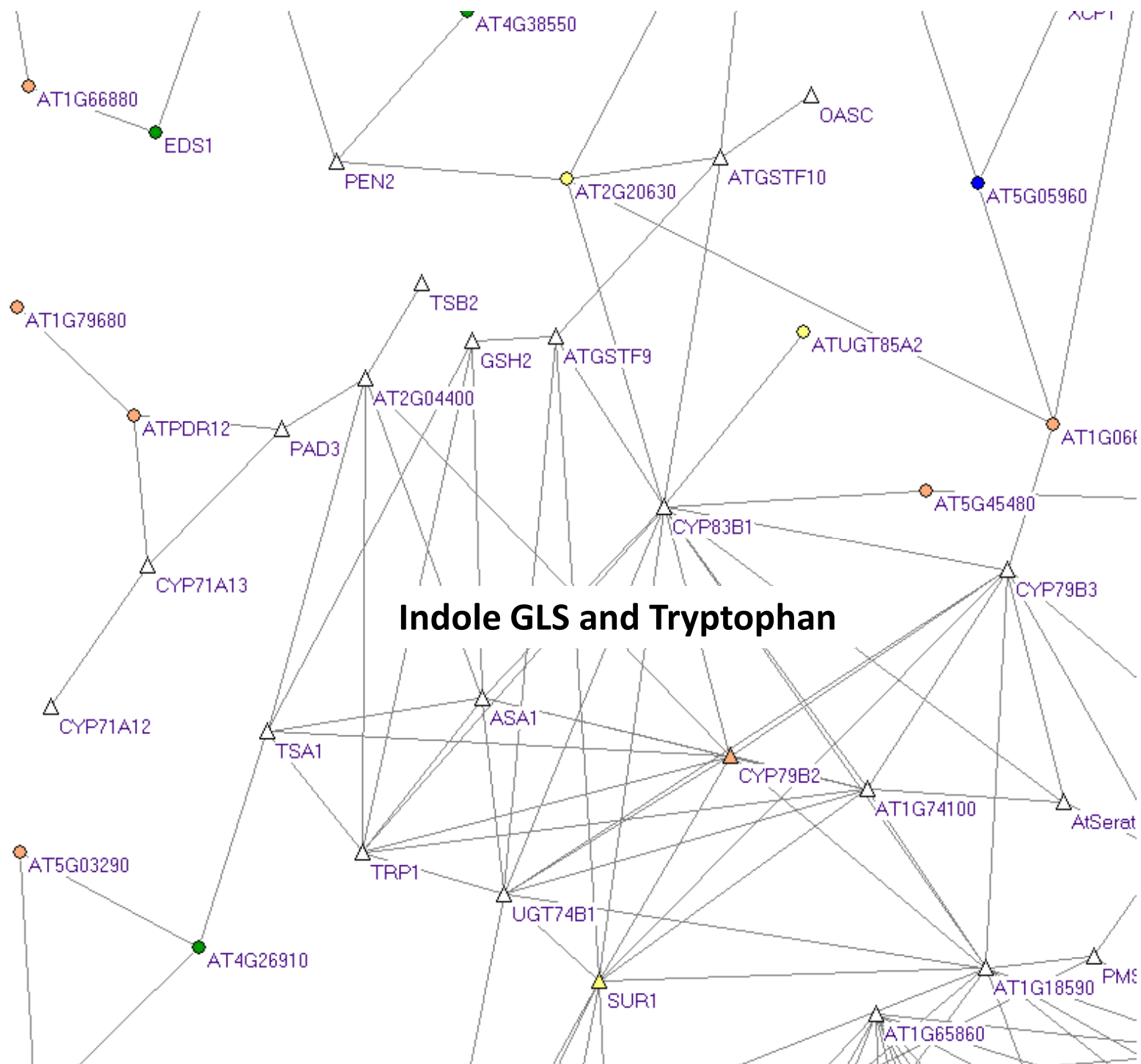

Supplement: Figure S3 — Core GSL co-expression network. The known or predicted GSL genes generate a core GSL co-expression network that is expanded in this presentation for legibility. The general biochemical functions of the four major clusters within this super network are labeled. Three of the major clusters are further magnified to provide gene identification. (PDF) [file pbio.1001125.s004.pdf]

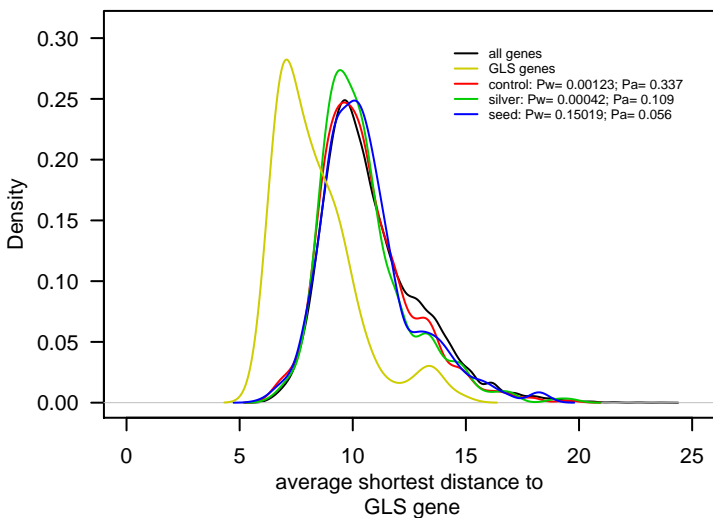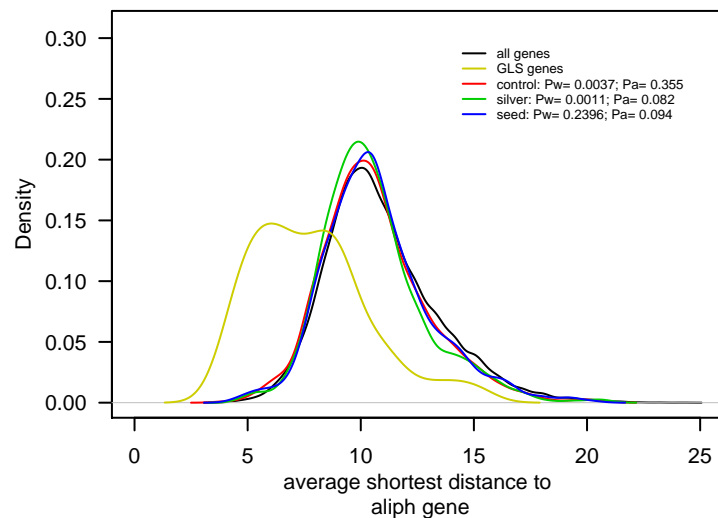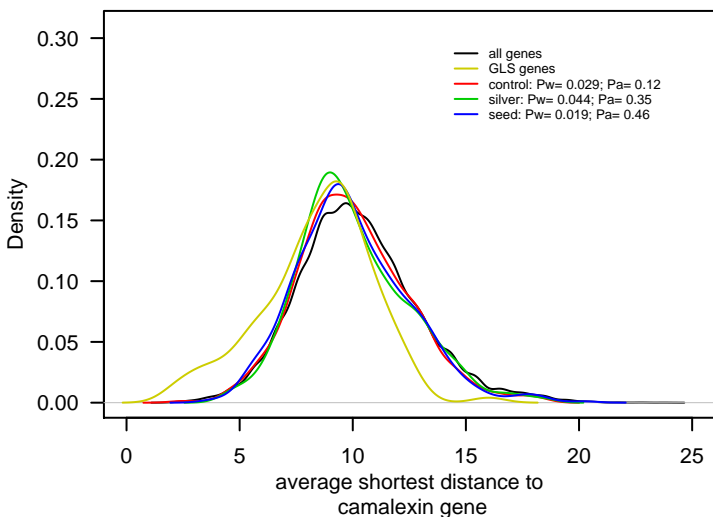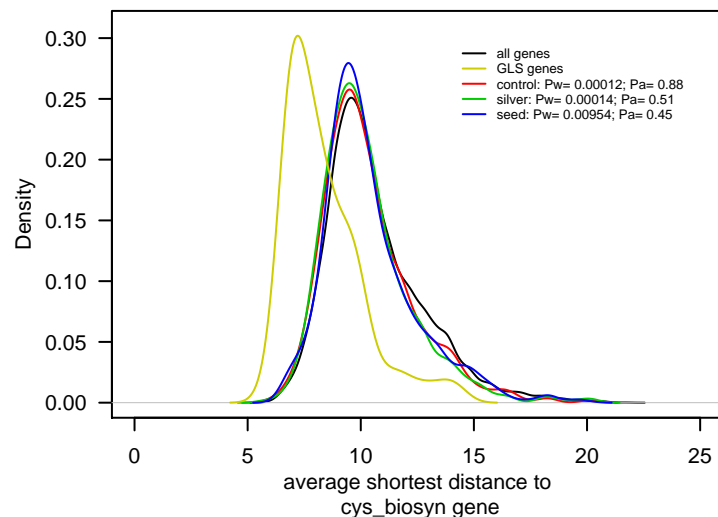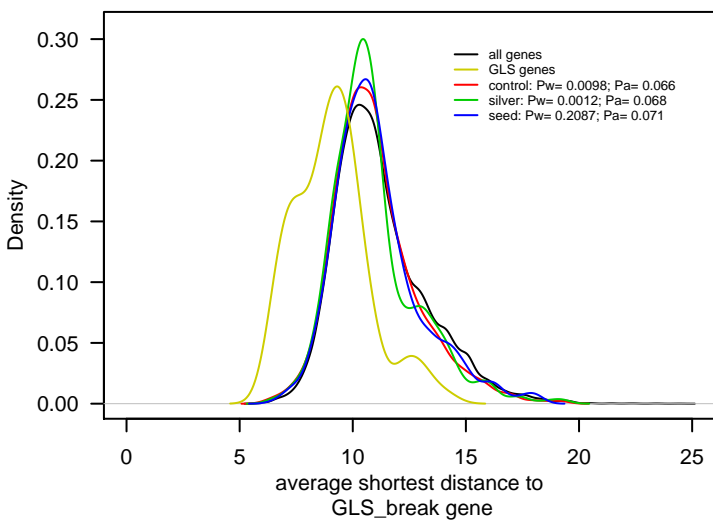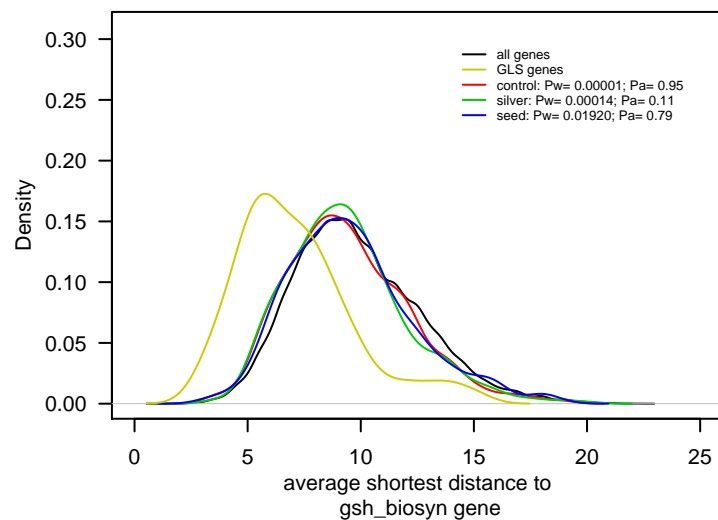

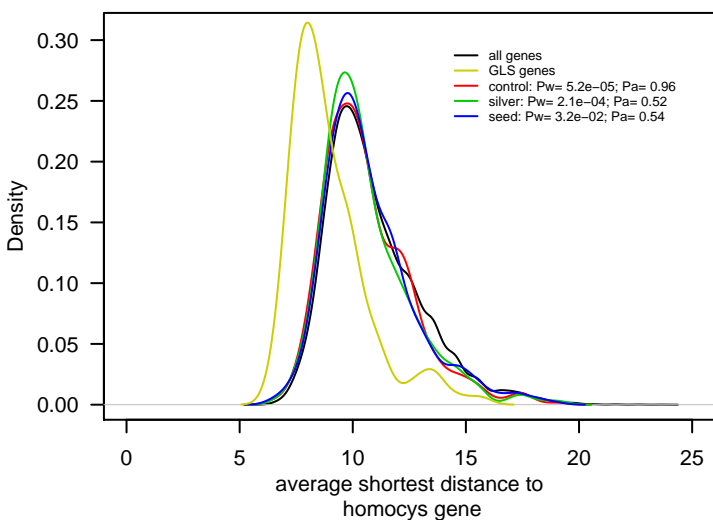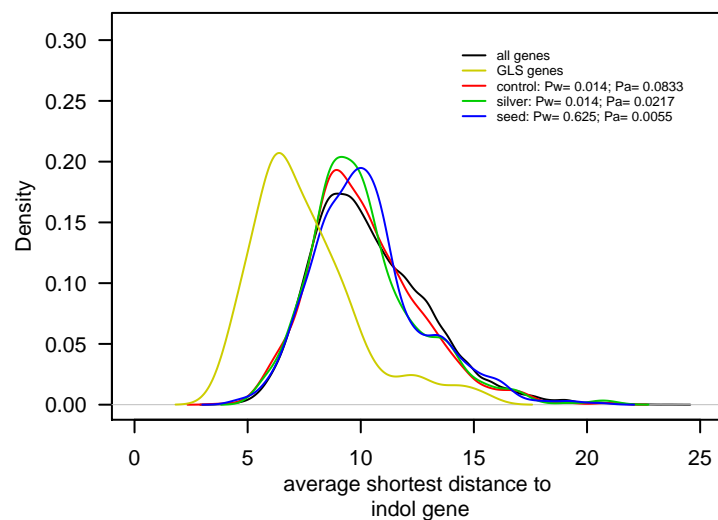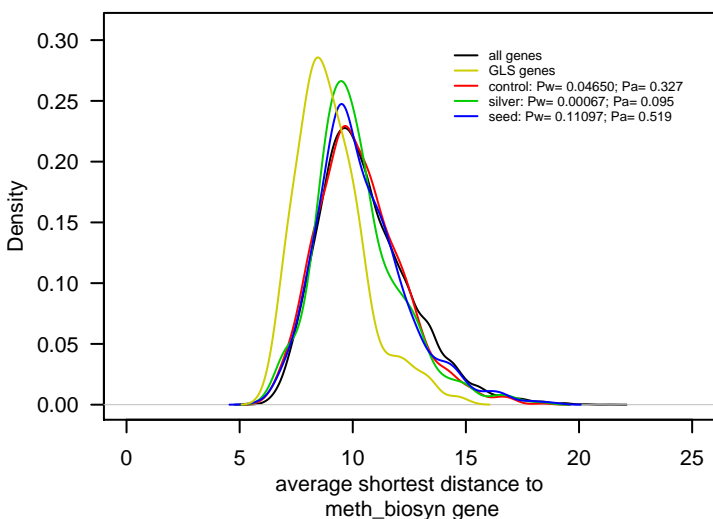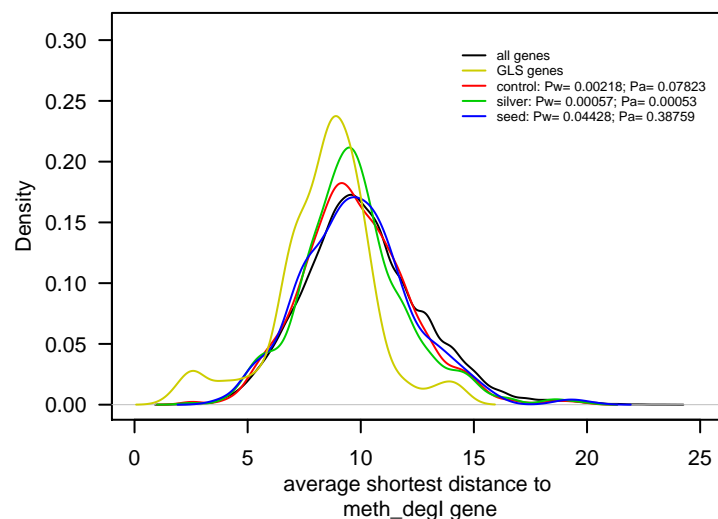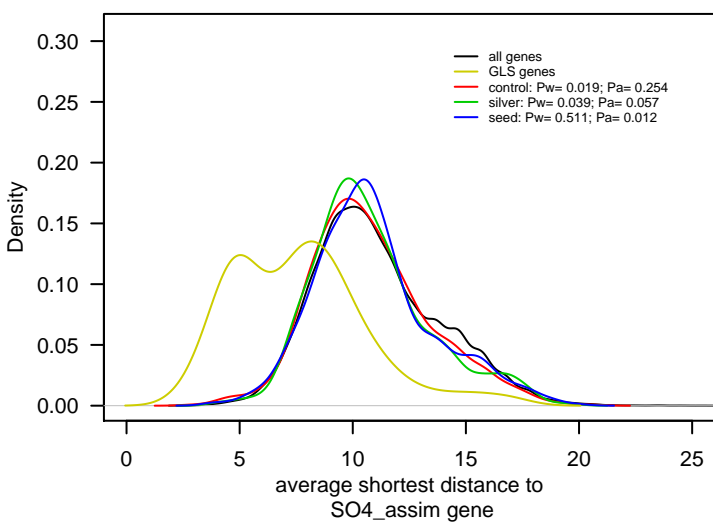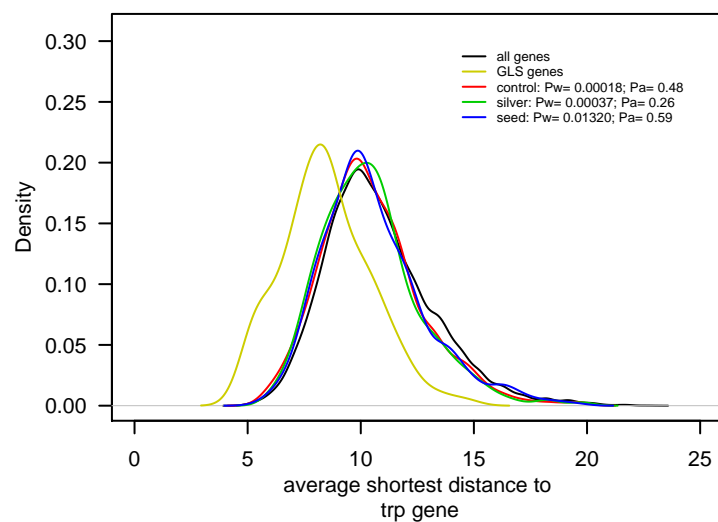

Supplement: Figure S4 — Distributions of shortest distances between known GSL genes and GWA candidates. Shown are plots comparing the distributions of the shortest distances between known GSL genes and GWA candidates for the control (red), silver (green), and seedling (blue) datasets. For comparison similar distributions derived from non-GWA-candidates (all genes) are also shown (black lines). Pw is the Wilcoxon Rank Sum test p value comparing the probability of a location shift between the distribution of the shortest paths of all GWA candidate genes (from one of the three datasets) to the corresponding glucosinolate gene and the distribution of the shortest paths of all non-significantly associated genes to the corresponding glucosinolate gene. Pa is the Ansari-Bradley Test probability assessing the difference in dispersion between the two aforementioned distributions. This was done for all GSL genes as well as for each of the specific biosynthetic networks as defined. (PDF) [file pbio.1001125.s005.pdf]
